# Effect of four fluoroquinolones on the viability of bladder cancer cells in 2D and 3D cultures

**DOI:** 10.3389/fonc.2023.1222411

**Published:** 2023-07-18

**Authors:** Tomasz Kloskowski, Zuzanna Fekner, Kamil Szeliski, Michelle Paradowska, Daria Balcerczyk, Marta Rasmus, Paweł Dąbrowski, Łukasz Kaźmierski, Tomasz Drewa, Marta Pokrywczyńska

**Affiliations:** ^1^ Chair of Urology and Andrology, Department of Regenerative Medicine, Collegium Medicum, Nicolaus Copernicus University, Bydgoszcz, Poland; ^2^ Chair of Urology and Andrology, Department of Tissue Engineering, Collegium Medicum, Nicolaus Copernicus University, Bydgoszcz, Poland

**Keywords:** bladder cancer, fluoroquinolones, enrofloxacin (ENR), moxifloxacin (MOX), ofloxacin (OFL), norfloxacin (NFX)

## Abstract

**Introduction:**

The anticancer properties of fluoroquinolones and the high concentrations they achieve in urine may help in bladder cancer therapy. This study aimed to analyze the properties of 4 fluoroquinolones as potential candidates for supportive treatment of bladder cancer.

**Methods:**

Comparative analyses were performed on the cytotoxic effects of norfloxacin, enrofloxacin, moxifloxacin, and ofloxacin on normal and cancer urothelial cell lines. In 2D culture, the cytotoxic properties of fluoroquinolones were evaluated using MTT assay, real-time cell growth analysis, fluorescence and light microscopy, flow cytometry, and molecular analysis. In 3D culture, the properties of fluoroquinolones were tested using luminescence assays and confocal microscopy.

**Results and Discussion:**

All tested fluoroquinolones in 2D culture decreased the viability of both tested cell lines in a dose- and timedependent manner. Lower concentrations did not influence cell morphology and cytoskeletal organization. In higher concentrations, destruction of the actin cytoskeleton and shrinkage of the nucleus was visible. Flow cytometry analysis showed cell cycle inhibition of bladder cancer cell lines in the G2/M phase. This influence was minimal in the case of normal urothelium cells. In both tested cell lines, increases in the number of late apoptotic cells were observed. Molecular analysis showed variable expression of studied genes depending on the drug and concentration. In 3D culture, tested drugs were effective only in the highest tested concentrations which was accompanied by caspase 3/7 activation and cytoskeleton degradation. This effect was hardly visible in non-cancer cell lines. According to the data, norfloxacin and enrofloxacin had the most promising properties. These two fluoroquinolones exhibited the highest cytotoxic properties against both tested cell lines. In the case of norfloxacin, almost all calculated LC values for bladder cancer cell lines were achievable in the urine. Enrofloxacin and norfloxacin can be used to support chemotherapy in bladder cancer patients.

## 1 Introduction

According to World Health Organization (WHO) estimates, cancer will be the leading cause of death and the most crucial obstacle to increasing life expectancy in every country during the 21^st^ century. It is estimated that up to 19.3 million new cases and 10.0 million deaths caused by cancer occurred in 2020 (1, 2). Bladder cancer is the 10^th^ most common type of cancer in the world and the 5^th^ in Europe, with an estimated 550,000 new cases annually. This cancer is more prevalent among men (6^th^) than women (18^th^). The incidence rates for both genders are highest in East Asia and Europe (1, 3, 4).

The most common method of bladder cancer treatment is surgery, such as transurethral resection of bladder tumor (TURBT) or cystectomy and are frequently combined with chemotherapy to prevent relapse and metastases. Unfortunately, the currently used methods are not effective enough, which is why, to avoid radical cystectomy, a new treatment protocol should be developed (5, 6).

Fluoroquinolones are chemotherapeutics and one of the largest groups of antibacterial agents used in the world. The mechanism of antibacterial action of fluoroquinolones is based on inhibiting the catalytic cycle of DNA gyrase and topoisomerase IV, enzymes that are responsible for the replication and transcription of bacterial DNA. Although fluoroquinolones have a greater affinity for bacterial topoisomerases, they also act similarly on the eukaryotic analogue - topoisomerase II. Quinolones destabilize DNA-splitting *via* topoisomerase II, which causes permanent DNA damage to the genome and consequently leads to apoptosis (7–9). This group of antibiotics shows excellent bioavailability after oral administration (30–80%) and a long half-life of up to 15 hours (9–12). The development of other generations led to improving their pharmacokinetic and pharmacodynamic properties while increasing their bactericidal activity against many clinically relevant pathogens, including Gram-negative, Gram-positive bacteria, and enterobacteria (9, 11). Fluoroquinolones penetrate tissues such as the bladder, prostate, kidney, and liver very well and are commonly used to treat various urinary and genital infections (8, 12).

Norfloxacin, ofloxacin, and enrofloxacin belong to the second generation of quinolones, and moxifloxacin to the fourth generation. We chose these drugs because, besides ciprofloxacin and levofloxacin, norfloxacin, ofloxacin, and moxifloxacin are the most frequently used fluoroquinolones in clinical practice (13, 14). Enrofloxacin is commonly used in veterinary medicine, and, in most species, is partially metabolized to ciprofloxacin, which may further contribute to its antibacterial action. The bioavailability of all fluoroquinolones after oral administration varies between 10–80% (15–19).

Many studies have been carried out to investigate fluoroquinolones’ anticancer properties on various cell lines. However, to our knowledge, comparative studies analyzing the cytotoxic effects of norfloxacin, enrofloxacin, moxifloxacin, and ofloxacin on both normal and cancer cell lines have not been performed so far. The effectiveness of these fluoroquinolones was, for the first time, analyzed in 3D culture. The anticancer properties of fluoroquinolones, their availability, and the high concentration that they achieve in the urine may help bladder cancer patients by supporting their chemotherapy (9, 15–17, 19–23).

## 2 Materials and methods

### 2.1 Cell culture

The human urinary bladder cancer cell line (T24) and human urothelium cell line (SV-HUC-1) were obtained from the American Type Culture Collection (ATCC, USA). The T24 cell line was cultured in Dulbecco’s Modified Eagle’s Medium (DMEM) Ham’s F-12 50/50 Mix (Corning, USA) and the SV-HUC-1 cell line in Kaighn’s Modification of Ham’s F-12 Medium (Corning, USA). Cells were kept at 37°C in a humidified atmosphere of 5% CO_2_. Both media were supplemented with 10% fetal bovine serum (FBS), 100 U/mL of penicillin-streptomycin, and 100 U/mL of amphotericin B (Corning, USA).

### 2.2 Drugs

Solutions ranging from 25 to 800 μg/mL were prepared by dissolving the Pharmaceutical Secondary Standard powders of moxifloxacin, ofloxacin, norfloxacin, and enrofloxacin (Sigma-Aldrich, USA) in the medium used for the appropriate cell line. Additionally, 0,05% 0,1M HCl (Reagecon, Ireland) was added to lower the pH to enable the achievement of higher solubility.

### 2.3 MTT assay

After detachment, 2.5 x 10^3^ T24 cells and 12.5 x 10^3^ SV-HUC-1 cells were seeded per well in a 96-well plate. The following day, different concentrations of moxifloxacin, ofloxacin, norfloxacin, or enrofloxacin were added (25, 50, 100, 200, 500, and 800 μg/mL), and after that, the cells were incubated for 24 and 48 hours at 37°C in 5% CO_2_. Untreated cells (with the acidified medium used for dissolving the drugs) were used as a control. After the incubation, 100 μL of 1-(4,5-Dimethylthiazol-2-yl)-3,5-diphenylformazan (MTT) reagent (1 mg/mL) was added to each well, and the plate was placed in the dark for two hours at 37°C. Next, the supernatant was removed, and dimethyl sulfoxide (DMSO, POCH, Poland) was added to dissolve formazan crystals. The absorbance was measured spectrophotometrically at 570 nm and 655 nm using a Varioscan LUX plate reader (ThermoFisher Scientific, USA). Obtained absorbance results were used to calculate cell viability after fluoroquinolone treatment and to calculate the lethal concentrations (LC) causing the death of 10, 50, and 90% of cells.

### 2.4 Real-time cell growth analysis

To confirm the results (LC_10_, LC_50_, and LC_90_) calculated based on the MTT assay, a real-time cell growth analysis was performed using the xCELLigence RTCA DP system (ACEA Bioscience, USA). This device uses data from the impedance measurement (electrical resistance) of biosensors placed at the bottom of each plate well to analyze changes in cell morphology and their proliferation rate. Analysis was performed on a 16-well E-plate (ACEA Bioscience, USA). Cells were seeded at a density of 5 x 10^3^ (T24) or 12 x 10^3^ cells per well (SV-HUC-1) and cultured in 200 μL of medium (37°C, 5% CO_2_ atmosphere). After 24 hours, previously prepared LC_10_, LC_50_, and LC_90_ concentrations of the appropriate antibiotics were added to the cells, and as a control, a fresh medium was used. The influence of fluoroquinolones on cell growth was analyzed for 24 and 48 hours.

### 2.5 Cell cycle analysis

Cell cycle changes were analyzed using flow cytometry and a dedicated Tali^®^ Cell Cycle Kit (Thermo Fisher Scientific, USA). After incubation with a fluoroquinolone, the cells were detached and centrifuged twice at 500 x g, and cells were washed with Phosphate Buffered Saline (PBS; Corning, USA) between stages. Next, 5 x 10^5^ cells were suspended in 1 mL of cold 70% ethyl alcohol (POCH, Poland) and incubated at -20°C for at least 24 hours. Then cells were again washed twice in PBS (Corning, USA) and centrifuged at 1000 x g at 4°C. The cells prepared this way were suspended in Tali^®^ Cell Cycle Kit solution (Thermo Fisher Scientific, USA) and incubated at room temperature in the dark for 30 minutes. Analysis was performed using the BD FACSCanto™ II flow cytometer (Becton Dickinson, United States). The obtained data were analyzed using FlowJo v10 (Becton, Dickinson, and Company, USA). The percentage of cells in the G0/G1, S, and G2/M phases was calculated using the Watson model.

### 2.6 Apoptosis detection

The test was performed using the FITC Annexin V Apoptosis Detection Kit II (BD PharmingenTM, USA), which consists of Annexin V conjugated with fluorescein isothiocyanate (FITC) and propidium iodide (PI). After 24 hours of exposure, to calculate LC values of the fluoroquinolones, cells were detached and washed with PBS (Corning, USA). Next, the cells were centrifuged at 500 x g, and 3.5 x 10^5^ cells were resuspended in 350 μL of binding buffer. Cells were stained with 5 μL of FITC-conjugated Annexin-V (10 mg/mL) and 10 μL of PI (50 mg/mL) and then incubated for 15 minutes at room temperature in the dark. After incubation, 200 μL of binding buffer was added, and cells were analyzed using a BD FACSCanto™ II flow cytometer (Becton Dickinson, USA). Results were analyzed with FACSDiva software (Becton Dickinson, USA).

The caspase activity was measured using the Caspace-Glo^®^3/7 assay (Promega, Walldorf, Germany). After seeding with the proper density on a 96-well white/clear plate (Corning, Germany), both cell lines were allowed to attach to the growth surface for 24 hours. Next, all the tested drugs’ LC concentrations calculated after 24 hours of incubation were added. Both reagents (substrate with buffer) were brought to room temperature, and after combining, an equal volume of reagent was added directly to cells in the culture medium. After that, the plate was placed in a Varioskan LUX plate reader (Thermo Fisher Scientific, USA), shaken for 30 seconds at 300 rpm, and incubated in the dark for 30 minutes, after which the luminescence signal was measured. The experiment was performed in triplicate.

### 2.7 Morphological analysis under light and fluorescent microscopy

For morphological analysis, T24 and SV-HUC-1 cell lines were seeded in a 12-wells plate on previously placed round glass slides at a concentration of 6 x 10^3^ and 15 x 10^3^ cells/well, respectively. The cells were treated with calculated LC concentrations of antibiotics for 24 and 48 hours. Morphological changes were examined under an inverted light microscope (DMi1, Leica, Germany).

After incubation, cells were washed with PBS and fixed with a 4% methanol-free formaldehyde solution in PBS containing 0.1% Triton-X100 (Sigma-Aldrich, USA). Next, a staining solution containing 4’,6-diamidino-2-phenyloindol (DAPI) at a concentration of 0.1 µg/mL and phalloidin-iFluor 488 (Sigma-Aldrich, USA) at a manufacturer-recommended concentration was added for 30 minutes. After that, three PBS washes were performed. Cells were visualized using an inverted fluorescence microscope (IX83, Olympus, Japan). Images were acquired in a z-stack, and deconvolution and EFI (extended focal imaging) were performed to obtain 2-dimensional images for publication.

### 2.8 Molecular analysis

Cells exposed to the tested fluoroquinolones at concentrations of LC_10_, LC_50_, and LC_90_ were analyzed for the expression of selected genes (*BAX*, *BCL2*, *TOP2A*, *TOP2B*, and *CDKN1A*). Total RNA from tested cells was purified using a RNeasy Mini Kit (Qiagen, Germany). The cell pellet was disrupted with an appropriate volume of lysis buffer and processed according to the manufacturer’s protocol. The obtained material was subjected to qualitative and quantitative analysis using the NanoDrop™ Lite Spectrophotometer (Thermo Fisher Scientific, USA). Reverse transcription reactions for complementary DNA synthesis were performed using the Transcriptor First Strand cDNA Synthesis Kit with the Anchored-oligo(dT)_18_ Primer (Roche, Switzerland) according to the manufacturer’s recommendations. Gene expression was evaluated by quantitative polymerase chain reaction (qPCR) using LightCycler^®^ 480 SYBR Green I Master (Roche, Switzerland) and PCR primers (Bio-Rad, USA) for selected genes with *SDHA* and *TBP* as the reference genes. The obtained gene expression results were compared to control cells and subjected to statistical analysis.

### 2.9 3D cell culture

Spheroids were generated using low-attachment plates. Cells were seeded at a density of 25,000 cells per well in 96-well U-shaped PrimeSurface^®^ 3D plates (PHC Europe, The Netherlands), centrifuged at 200 x g for 5 minutes, and grown for 3 to 4 days. Transparent plates were used for morphology and white for luminescence analyses. CellTiter-Glo^®^ 3D assay (Promega, Germany) was used for the measurement of the spheroids viability. Calculated LC concentrations of tested drugs were added to the spheroids and incubated for 24 and 48 hours. The reagent was placed in a refrigerator for the night, and the next day it was prewarmed (22°C, for 30 minutes) directly before use. Fifty microliters of reagent were added to the same volume of culture medium. After that, the plate was placed in a Varioskan LUX plate reader (Thermo Fisher Scientific, Massachusetts, USA), shaken for 5 minutes at 420 rpm, and incubated in the dark for 30 minutes, after which the luminescence signal was measured. Caspase-Glo^®^3/7 3D assay (Promega, Germany) was used for caspase activity measurement. To prepare the reagent for analysis, the substrate was combined with a buffer and left at room temperature before the experiment. The protocol was similar to the viability assay except for the shaking parameters (30 seconds at 500 rpm). Each experiment was performed in triplicate. The light-inverted microscope, Leica DMi1 (Leica, Germany), was used for photographic documentation. The spheroids area was measured using EPview 1.3 software (Olympus, Japan).

In the case of cytoskeleton analysis, before staining, spheroids were washed with PBS and permeabilized using 0.1% Triton-X100 for 24 hours at 4°C. F-actin was stained using Phalloidin-iFluor™ 647 (concentration following the manufacturer’s specification; Cayman Chemical, USA), and the nuclei were stained with DAPI (600 nM) for over 4 hours in 4°C. The 647 marker was used to increase sample penetration. After staining, spheroids were washed with PBS twice and transferred from their wells onto a dedicated imaging dish for confocal microscopy (µ-Dish 35 mm, high Glass Bottom; ibidi, USA). Two different imaging approaches were used to inspect the changes on a macro and micro-scale thoroughly. For the investigation of the spheroid structure, a 10x lens was used. For a more detailed examination of the cytoskeleton of the cells comprising the spheroid, a narrower field of view and higher resolution, a 63x lens, was more appropriate (LSM 900, Zeiss, Germany). For the images acquired by the 10x lens, the dedicated ZEN software deconvolution was applied. In the case of the higher magnification, airyscan software processing was performed in the workflow.

### 2.10 Statistical analysis

Each experiment was performed at least in triplicate. Average cell viability was expressed as a percentage relative to the control. All data were presented as means ± SD (Standard Deviation). Normality distribution was analyzed using the Shapiro-Wilk test. Statistical analysis was performed using a one-way ANOVA with Tukey *post hoc* (for cell viability) or two-way ANOVA with Sidak *post hoc* (for grouped analysis). Kruskal-Wallis with Dunn’s multiple comparison tests were used for results with non-normal distributions. Pearson comparison was performed to compare the cytotoxicity profiles of the tested drugs on all tested cell lines (GraphPad Software 8.4., USA).

## 3 Results

### 3.1 Fluoroquinolones reduce T24 and SV-HUC-1 cell viability

**Figure 1:**
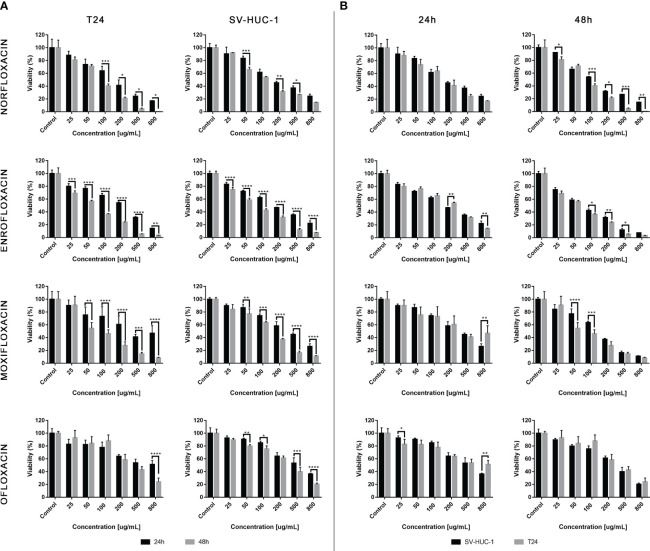
Fluroquinolones cytotoxicity. The effect of norfloxacin, enrofloxacin, moxifloxacin, and ofloxacin on the viability of human bladder cancer cells (T24) and human urothelium cells (SV-HUC-1). Cells were treated with various concentrations of fluoroquinolone (25-800 μg/mL) for 24 and 48h and examined using the MTT assay. **(A)** – comparison between incubation times; **(B)** – comparison between normal and cancer cells. Data are expressed as % of the controls. Statistical significance was presented as * p< 0.05; ** p< 0.01; *** p <0.001; **** p<0.0001.

The results showed that all antibiotics significantly reduce cell viability depending on dose and incubation time (**Figure 1**). In most cases, cell viability after 48 hours of incubation was lower compared to 24 hours of incubation (**Figure 1A**). The concentration dependence on cell survival in all cases was logarithmic. Based on the obtained trend lines, a formula was generated, which enabled the calculation of the LC_10_, LC_50_, and LC_90_ values (**Table 1**, **Supplementary Table 1**). Higher cytotoxicity to bladder cancer than to non-cancer urothelium cells was observed after 48 hours of incubation in the case of norfloxacin at almost all of the concentrations and enrofloxacin at the highest tested concentration. In two other tested drugs, differences between normal and cancer cell lines were observed in single concentrations (**Figure 1B**). Norfloxacin and enrofloxacin showed the strongest anticancer properties against cancer cell lines and weaker effects on normal cells. Calculated LC_90_ values after 48-hour incubation of SV-HUC-1 were 2 times higher than for T24 cells in the case of norfloxacin, which means that non-cancer urothelium cells are more resistant to this antibiotic. Despite the lowest cytotoxic potential of all tested drugs, ofloxacin also shows a high potential due to significant differences between LC_90_ values for T24 cells (LC_90 = _6364.7 μM) and SV-HUC-1 (LC_90 = _12386.3 μM) after 24-hour incubation with the drug.

**Table 1:** Calculated LC values (μM) after T24 and SV-HUC-1 cell lines incubation with norfloxacin, enrofloxacin, moxifloxacin, and ofloxacin for 24 and 48 hours.

		NORFLOXACIN		ENROFLOXACIN		MOXIFLOXACIN		OFLOXACIN	
	[μM]	T24	SV-HUC-1	T24	SV-HUC-1	T24	SV-HUC-1	T24	SV-HUC-1
**24h**	**LC_10_**	66.3	83.2	62.3	51.8	49.8	74.7	290.6	116.2
	**LC_50_**	457.6	564.1	500.5	516.3	1071.2	749.8	1917.7	1195.5
	**LC_90_**	3160.3	5141.7	4019.3	5143.4	5729.5	7577.9	6364.7	12386.3
**48h**	**LC_10_**	40.2	60.2	22.5	28.6	15.0	52.3	121.8	63.7
	**LC_50_**	226.8	404.4	168.2	220.2	171.9	316.4	935.3	600.5
	**LC_90_**	1281.6	2716.9	1255.0	1694.9	1606.8	1923.1	3163.0	4662.8

### 3.2 Morphological changes of tested cells after fluoroquinolone treatment

As shown in **Supplementary Figure 1**, untreated cells grew adhesively in the plate wells and were regular in shape and size. Treatment with LC_10_ doses did not affect the morphology of both cancer and normal cells (**Supplementary Figure 1**). A significant decrease in the number of cells and loss of contact between the cells was observed at LC_50_ concentrations. The cells treated with LC_90_ doses of fluoroquinolones for 24 and 48 hours lost their shape, became round, and began to detach from the well’s surface (**Supplementary Figure 1**). Additionally, at the LC_90_ concentration of norfloxacin and enrofloxacin crystals of these drugs started to precipitate (**Supplementary Figure 1**).

### 3.3 Real-time growth cell analysis confirmed the results of the MTT assay

Real-time cell growth of SV-HUC-1 and T24 lines after incubation with norfloxacin, enrofloxacin, moxifloxacin, and ofloxacin was performed using the xCELLigence RTCA system. The results confirmed that the LC values of these antibiotics, calculated based on the MTT test results, caused a decrease in cell proliferation by 10, 50, and 90% (**Supplementary Figure 2**).

### 3.4 Cell cycle analysis showed G2/M arrest in cancer cells

**Figure 2:**
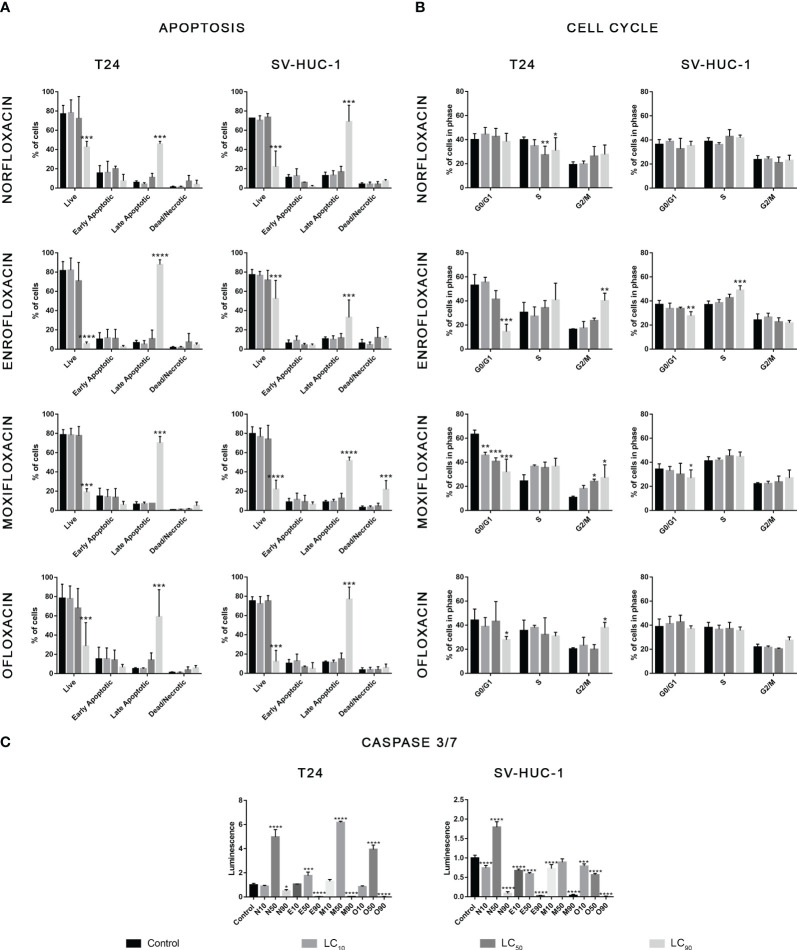
Results of apoptosis and cell cycle analysis. T24 and SV-HUC-1 cells after 24h incubation with LC_10_, LC_50_, LC_90_ of norfloxacin (N), enrofloxacin (E), moxifloxacin (M), and ofloxacin (O). Incubation with fluoroquinolones induced an increase of late apoptotic cells in both tested cell lines **(A)**, G2/M arrest in T24 cell line **(B)**, and activation of caspases 3/7 after treatment with LC_50_ concentration mainly in cancer cells **(C)**. SV-HUC-1 - normal human urothelium; T24 - human bladder cancer. Statistical significance was presented as * p< 0.05; ** p< 0.01; *** p <0.001; **** p<0.0001.

In the case of the T24 cell line, an increase in the number of cells in the G2/M phase was observed with a simultaneous decrease in the number of cells in the G1/G0 phase after exposure to all tested drugs, mainly at the LC_90_ concentrations. However, statistically significant values were not obtained in the case of norfloxacin. This result indicates that the analyzed fluoroquinolones induce G2/M phase arrest in T24 cells. In the case of the SV-HUC-1 cell line, the effect of the tested drugs on cell cycle phase distribution was minimal. We observed an increase in the number of cells in the S phase and a simultaneous decrease in the number of cells in the G1/G0 phase after exposure to enrofloxacin. Also, a reduction in G1/G0 phases after exposure to moxifloxacin was seen. In both cases, these findings were seen after using the LC_90_ concentration (**Table 2**, **Figure 2B**).

**Table 2:** Results (in percentage) of cell death type analysis of T24 and SV-HUC-1 cell line after exposure to LC_10_, LC_50_, and LC_90_ norfloxacin, enrofloxacin, moxifloxacin, and ofloxacin for 24 hours.

		T24				SV-HUC-1			
		AnV-/PI-	AnV+/PI-	AnV+/PI+	AnV-/PI+	AnV-/PI-	AnV+/PI-	AnV+/PI+	AnV-/PI+
**NORFLOXACIN**	**Control**	77.2 ± 8.8	15.6 ± 7.7	6.0 ± 1.6	1.2 ± 0.8	72.6 ± 0.1	10.9 ± 3.2	12.8 ± 3.7	3.9 ± 1.6
	**LC_10_**	78.4 ± 13.3	16.3 ± 11.6	4.1 ± 1.5	1.1 ± 0.8	70.4 ± 4.8	12.6 ± 7.4	13.2 ± 4.9	3.8 ± 2.4
	**LC_50_**	72.5 ± 22.7	20.2 ± 2.6	11.1 ± 4.2	7.3 ± 5.8	73.5 ± 4.0	5.7 ± 0.5	16.8 ± 5.9	3.9 ± 2.8
	**LC_90_**	42.7 ± 5.9	7.5 ± 6.7	45.9 ± 2.7	4.0 ± 4.2	22.1 ± 16.4	1.6 ± 1.1	69.0 ± 17.1	7.3 ± 1.5
**ENROFLOXACIN**	**Control**	81.5 ± 9.4	10.3 ± 6.9	6.6 ± 2.5	1.5 ± 1.2	77.2 ± 5.6	6.1 ± 3.4	10.6 ± 2.3	6.1 ± 4.0
	**LC_10_**	82.1 ± 12.7	11.6 ± 9.0	5.1 ± 3.8	1.6 ± 1.2	76.6 ± 4.2	8.8 ± 4.9	10.1 ± 3.8	4.6 ± 2.8
	**LC_50_**	70.8 ± 19.3	11.1 ± 9.4	10.7 ± 9.1	7.3 ± 9.0	71.6 ± 10.2	4.3 ± 1.8	11.5 ± 4.7	12.0 ± 2.0
	**LC_90_**	5.4 ± 2.1	2.4 ± 1.5	87.9 ± 5.0	4.5 ± 1.9	52.5 ± 18.9	3.8 ± 1.6	32.9 ± 18.3	11.2 ± 2.0
**MOXIFLOXACIN**	**Control**	78.6 ± 5.5	14.8 ± 8.3	6.2 ± 2.9	0.6 ± 0.3	79.7 ± 7.3	8.6 ± 3.9	8.6 ± 1.8	2.9 ± 1.7
	**LC_10_**	78.4 ± 6.9	14.0 ± 7.6	6.7 ± 1.9	1.0 ± 0.3	76.5 ± 9.1	11.2 ± 6.8	9.2 ± 2.4	3.2 ± 1.6
	**LC_50_**	77.6 ± 9.6	13.6 ± 9.1	7.3 ± 0.0	1.2 ± 0.8	74.0 ± 14.5	8.8 ± 6.9	12.6 ± 5.1	4.4 ± 3.2
	**LC_90_**	18.9 ± 3.6	5.8 ± 3.6	70.2 ± 6.6	4.9 ± 3.8	21.9 ± 9.6	6.3 ± 2.3	51.7 ± 3.6	21.8 ± .9.2
**OFLOXACIN**	**Control**	78.3 ± 14.7	15.8 ± 12.5	5.1 ± 1.2	1.0 ± 0.8	75.2 ± 4.4	10.3 ± 4.0	11.3 ± 1.5	3.4 ± 2.4
	**LC_10_**	77.8 ± 13.3	15.2 ± 11.5	5.2 ± 0.9	1.0 ± 0.6	72.3 ± 7.5	12.7 ± 7.3	11.2 ± 2.5	3.8 ± 2.5
	**LC_50_**	67.9 ± 20.5	14.0 ± 10.4	14.1 ± 7.4	3.8 ± 3.2	75.2 ± 5.6	6.3 ± 1.3	14.7 ± 6.3	3.7 ± 3.1
	**LC_90_**	28.6 ± 24.4	6.5 ± 3.0	59.2 ± 28.1	5.7 ± 2.7	12.2 ± 11.6	4.9 ± 6.2	77.1 ± 12.6	5.9 ± 3.7

AnV-/PI-: Alive; AnV+/PI-: probably early apoptotic; AnV+/PI+: Dead/late apoptotic/necrotic, AnV-/PI+: membrane-free nuclei, where with a decrease of PI signal, degradation of genetic material is observed. AnV – annexin-V; PI – propidium iodide.

In both tested cell lines, incubation with each of the four tested fluoroquinolones in LC_90_ concentrations increased the number of late apoptotic cells. In the other two tested concentrations, no changes in apoptotic cell distribution were observed (**Table 2**, **Figure 2A**). In the cancer cell lines, activation of caspase 3/7 was observed after incubation with a LC_50_ of all tested drugs. In the case of the SV-HUC-1 cell line, an increase in caspase 3/7 activation was observed only in the norfloxacin LC_50_ group (**Figure 2C**).

### 3.5 Fluoroquinolones induced shrinkage of the cell cytoskeleton

**Figure 3:**
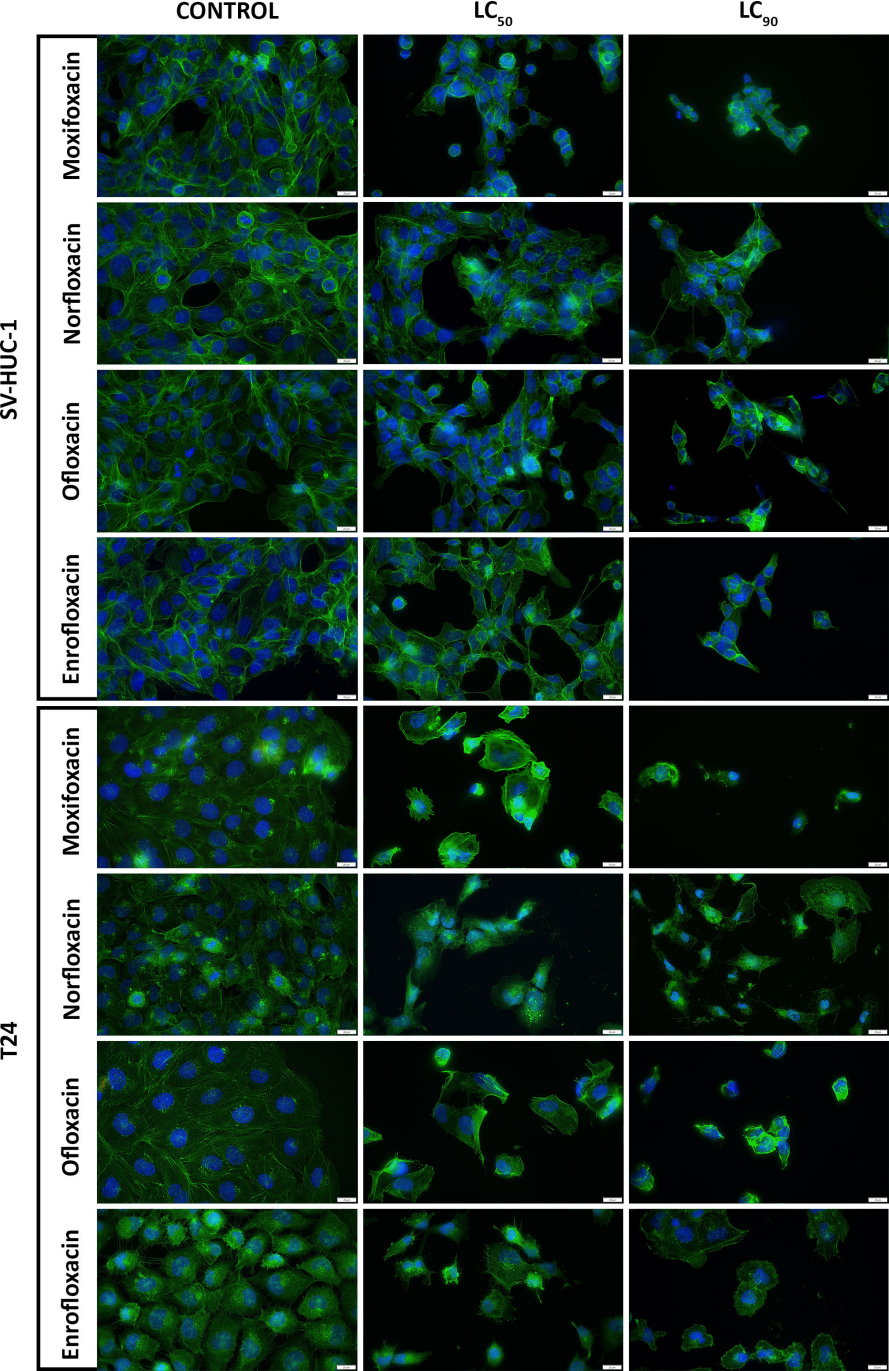
Immunofluorescence staining of cells cytoskeleton. Results of actin cytoskeleton visualization after exposure of T24 and SV-HUC-1 cells to LC_50_ and LC_90_ concentrations of norfloxacin, enrofloxacin, moxifloxacin, and ofloxacin for 24 hours. Decreases in cell number, cell shrinkage, and cytoskeletal degradation are visible. Fluorescence inverted microscope, bar=75 µm. SV-HUC-1 - normal human urothelium; T24 - human bladder cancer.

For all tested cell lines, the LC_90_ of the drugs showed severe cytoskeleton degradation and a decrease in the number of cells visible in the field of view compared to the control. In most cases, it was impossible to observe F-actin stress fibers due to severe degradation, with norfloxacin in the SV-HUC1 cell line being the exception. LC_90_ of ofloxacin and moxifloxacin was also responsible for severe morphological cell nucleus changes (**Figure 3**; **Supplementary Figure 3**).

Both LC_90_ and LC_50_ of all tested drugs decreased the staining efficiency of phalloidin. Staining uniformity was hindered, and the resulting signal was weaker compared to controls (data not shown). LC_10_ of ofloxacin and norfloxacin had the least effect on the morphological changes of the SV-HUC1 cell line, while norfloxacin had the least impact on T24 cells. Moxifloxacin at all tested concentrations caused the detachment of the remaining T24 cells and hindered the fixation process. Similar observations were made for T24 cells exposed to LC_90_ of ofloxacin (**Figure 3**; **Supplementary Figure 3**).

### 3.6 Similar cytotoxic profile of all tested fluoroquinolones

**Figure 4:**
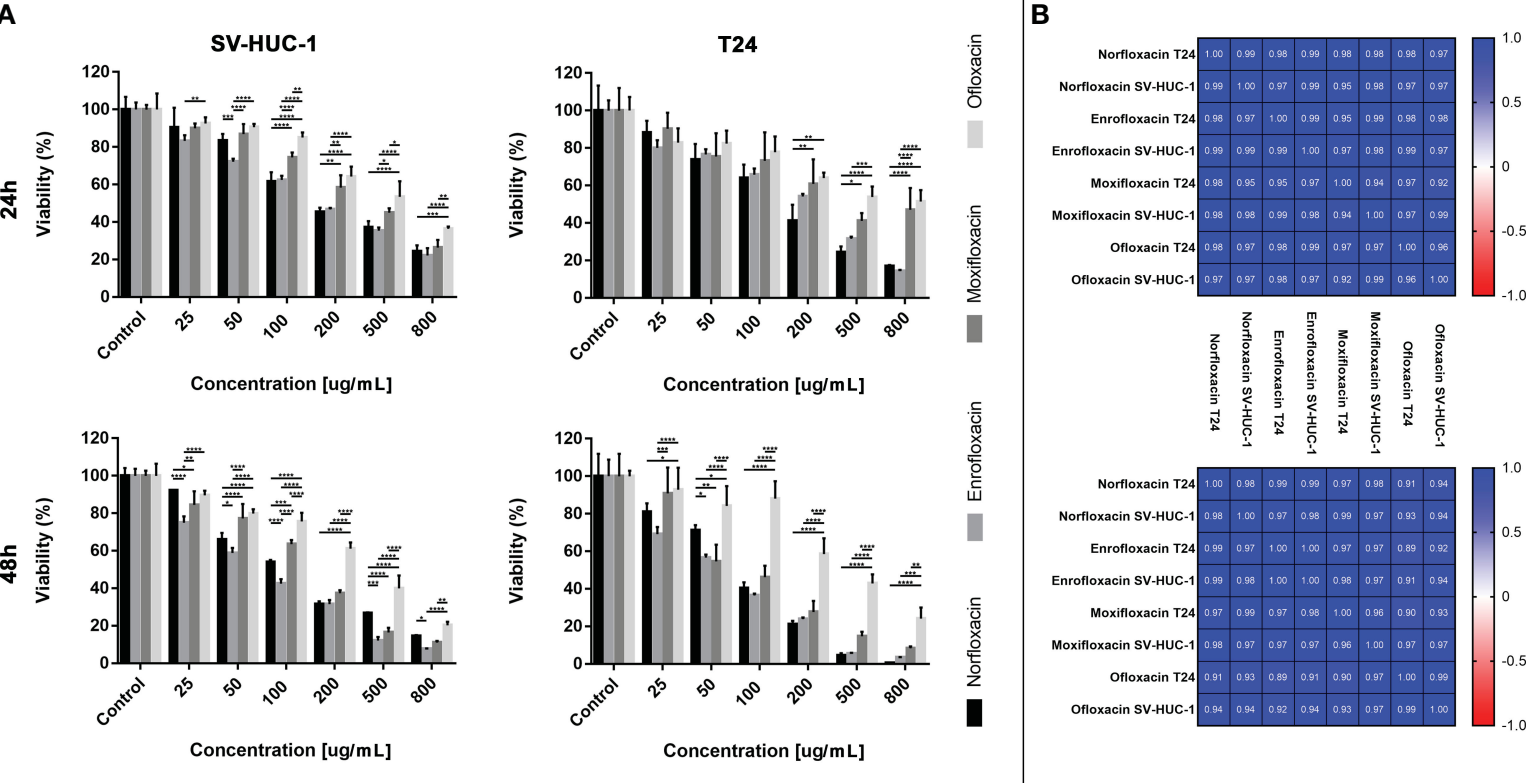
Fluoroquinolones effectiveness. **(A)** - Comparison of cytotoxic properties between all four tested fluoroquinolones. **(B)** - Pearson comparison of cytotoxic profiles of four drugs on normal and cancer cell lines. Results were obtained using MTT assay after 24 and 48 hours of incubation with both drugs. Norfloxacin and enrofloxacin were more effective against cancer cell line. SV-HUC-1 - normal human urothelium; T24 - human bladder cancer; * p< 0.05; ** p< 0.01; *** p <0.001; **** p<0.0001.

The cytotoxic profile analysis of all tested fluoroquinolones showed similar effects on both tested cell lines. The most significant differences were observed in the case of ofloxacin, which showed the weakest effect, especially against the T24 cell line, after 48 hours of incubation. Moxifloxacin also showed a weaker effect, however, its cytotoxic profile in higher doses was more comparable to norfloxacin and enrofloxacin than ofloxacin. In the case of norfloxacin and enrofloxacin, differences were rarely observed and mostly with low doses of drugs. However, after the 48-hour incubation, enrofloxacin was more effective in reducing non-cancer cell line viability (**Figure 4A**). These observed results were confirmed by Pearson comparisons of the cytotoxic profiles. Correlation between all tested drugs was very high, almost all combinations reached values above 0.9. This analysis showed a very high similarity of cytotoxic profiles between the tested drugs in both incubation times. The lowest value, below 0.9, was observed in the case of ofloxacin, which confirmed previous results indicating the insufficient effectiveness of this drug (**Figure 4B**).

### 3.7 Molecular analysis showed different patterns of gene expression

**Figure 5:**
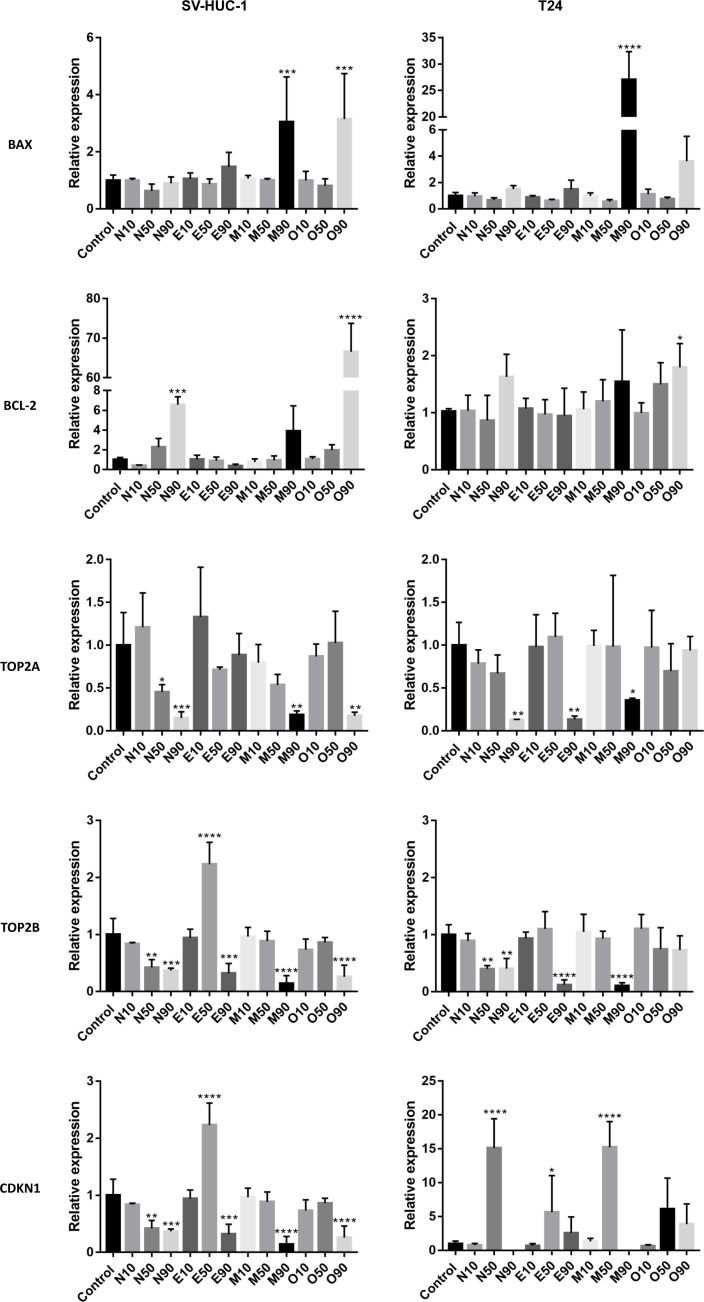
Molecular analysis of gene expression. The experiment was performed using LC values of norfloxacin (N), enrofloxacin (E), moxifloxacin (M), and ofloxacin (O) calculated after 24h incubation. Relative gene expression of control was calculated as 1. Variable expression of studied genes, depending on drug and concentration, was observed. SV-HUC-1 - normal human urothelium; T24 - human bladder cancer; * p< 0.05; ** p< 0.01; *** p <0.001; **** p<0.0001.

Overexpression of the *BAX* gene was observed in the highest tested concentration of moxifloxacin and ofloxacin in both cell lines. This effect was evident in the case of moxifloxacin in the T24 cell line. The *BCL-2* gene was overexpressed in non-cancerous cell lines after treatment with a LC_90_. In the case of cancer cells, only a slight increase was observed in the LC_90_ ofloxacin group. Tested drugs reduced the expression of *TOP2A* and *TOP2B* genes at the highest concentration. This effect was not observed in the case of ofloxacin in the T24 cell line and enrofloxacin for the *TOP2A* gene in the SV-HUC-1 cell line. In the *CDKN1* gene, we observed an increased expression with the LC_50_, mainly in cancer cells, and a decrease in its expression in LC_90_ (mainly in non-cancer cells). In the SV-HUC-1 cell line treated with LC_90_ of norfloxacin and moxifloxacin, the product of this gene was not detected (**Figure 5**).

### 3.8 The 3D assay showed the advantage of fluoroquinolones over cancer cells

**Figure 6:**
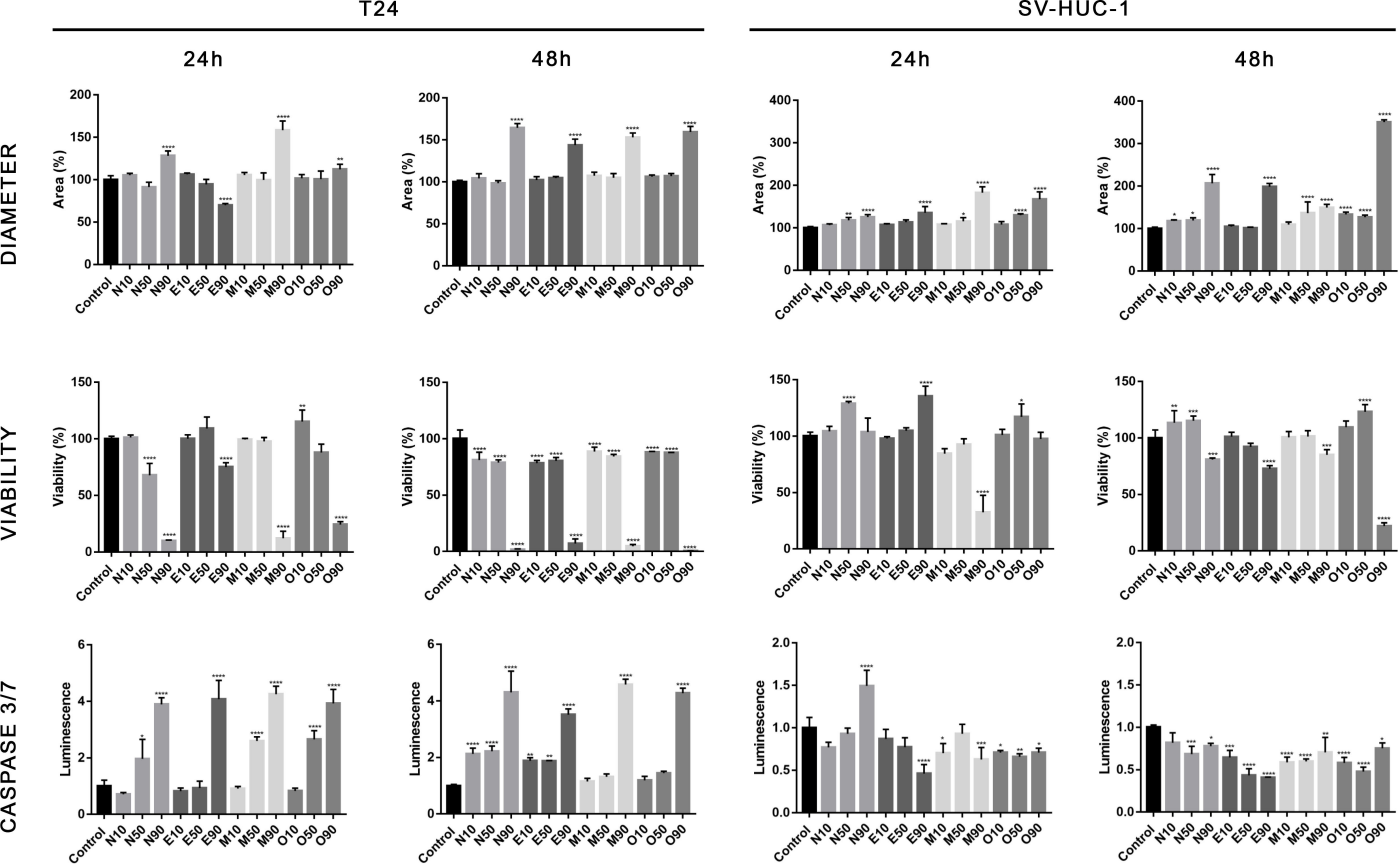
Fluoroquinolones’ effectiveness in 3D culture. Analysis of spheroids diameter, cell viability, and activity of caspase 3/7. Results were obtained using luminescence assays after 24 and 48 hours of incubation with LC values of norfloxacin (N), enrofloxacin (E), moxifloxacin (M), and ofloxacin (O). An increase in spheroids diameter, decrease in viability, and activation of caspases 3/7 was observed after treatment with LC_90_ concentration, especially in cancer cells. SV-HUC-1 - normal human urothelium; T24 - human bladder cancer; * p< 0.05; ** p< 0.01; *** p <0.001; **** p<0.0001.

**Figure 7:**
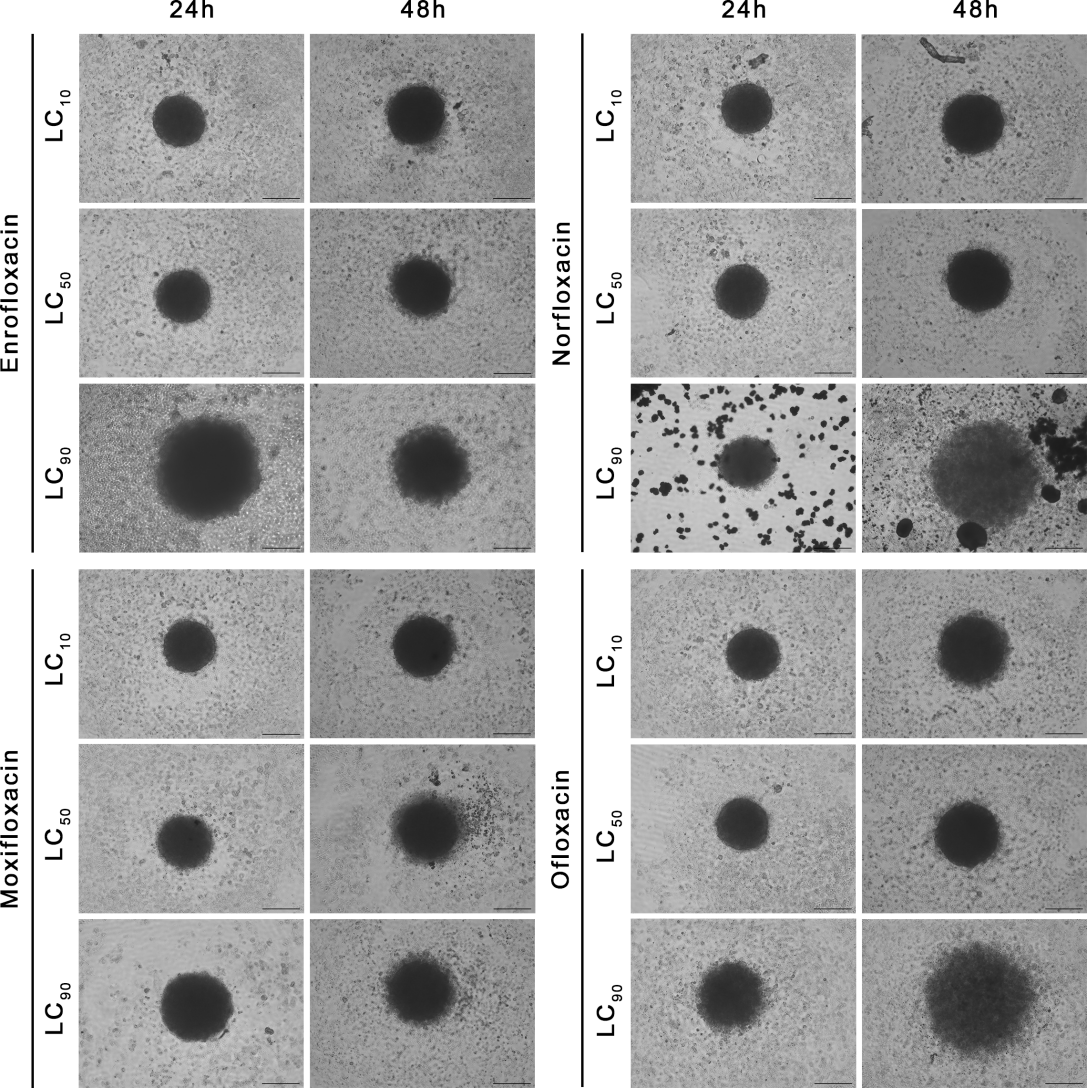
Spheroids morphology in normal human urothelium cell line (SV-HUC-1). Spheroids were incubated with LC concentrations of tested drugs. Enlargement in diameter can be observed in the highest tested concentration (LC_90_). Inverted light microscope; bar=200 µm.

**Figure 8:**
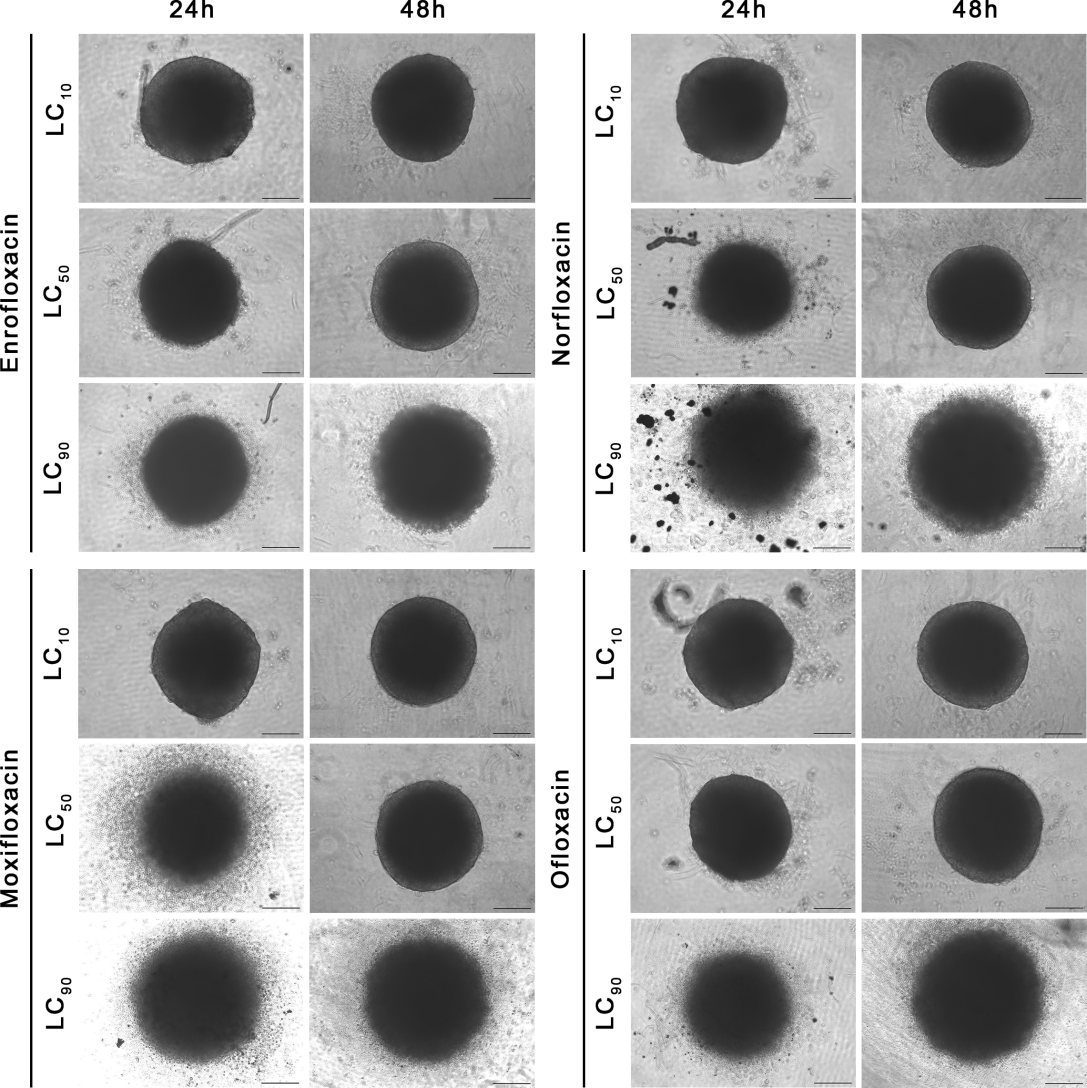
Spheroids morphology in human bladder cancer cell line (T24). Spheroids were incubated with LC concentrations of tested drugs. Enlargement in diameter can be observed in the highest tested concentration (LC_90_). Inverted light microscope; bar=200 µm.

The cytotoxic effect of fluoroquinolones was more pronounced in cancer cell lines in 3D cultures. This effect was visible, especially with the LC_90_ in T24 cell lines after both incubation times, except for the LC_90_ of enrofloxacin after a 24-hour incubation, in which cell viability was higher (**Figure 6**). Together decreased viability and increased spheroid diameter and caspase 3/7 activity was observed (**Figures 6**–**8**). LC_50_ did not reduce cell viability by 50% in 3D cultures. In the case of the SV-HUC-1 cell line, the cytotoxic effect was visible only in the LC_90_ moxifloxacin group after 24 hours and LC_90_ ofloxacin after 48 hours (**Figure 6**).

**Figure 9:**
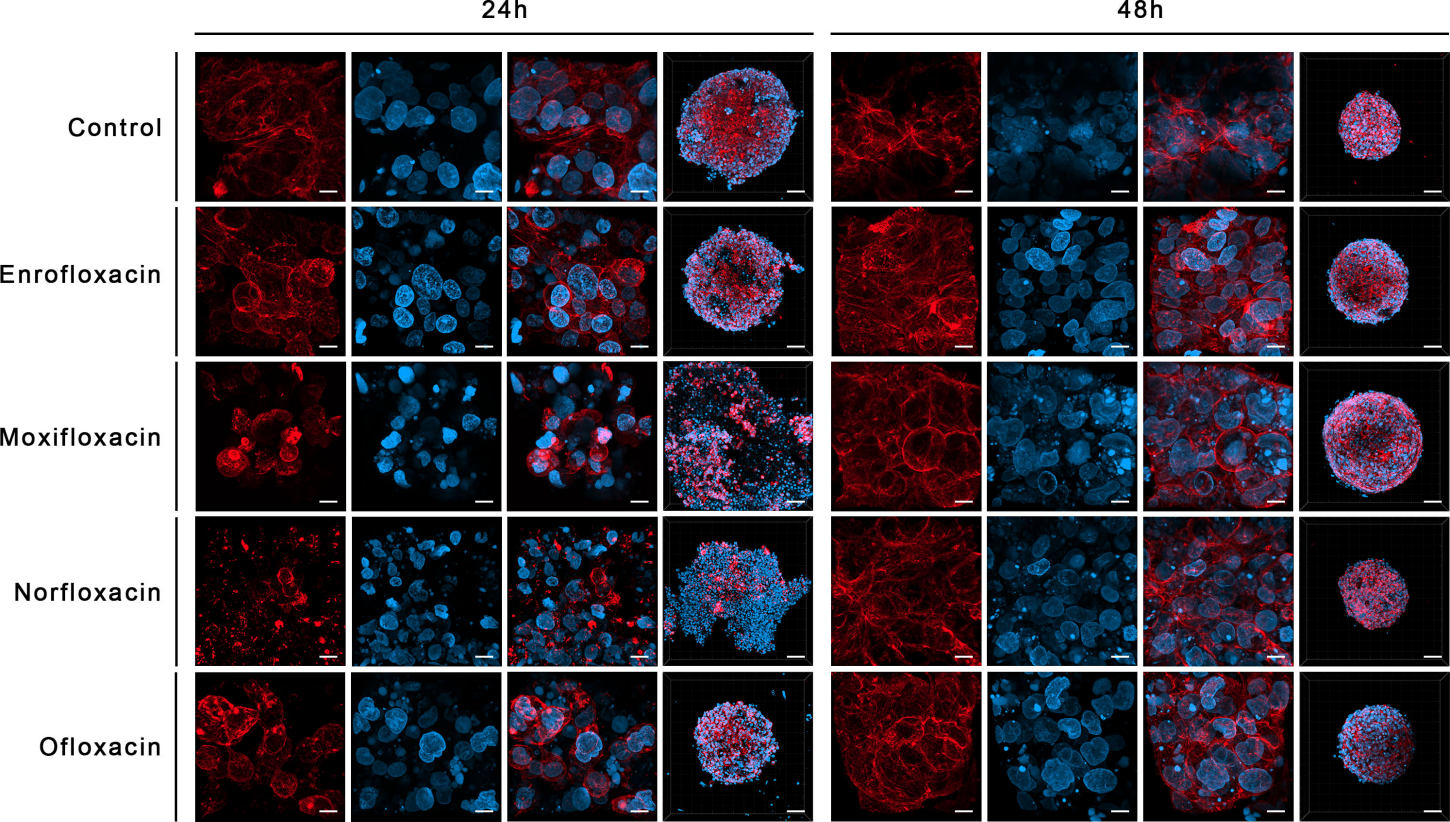
Immunofluorescence staining of cells cytoskeleton in 3D culture in a non-cancer human urothelium cell line (SV-HUC-1). Results of actin cytoskeleton visualization after exposure cells to LC_90_ concentrations of tested drugs for 24 and 48 hours. The affection of structural integrity, changes in cytoskeletal organization, and nucleus appearance can be observed. Confocal microscope, bar=80 µm and 10 µm for 10x and 63x magnification, respectively.

**Figure 10:**
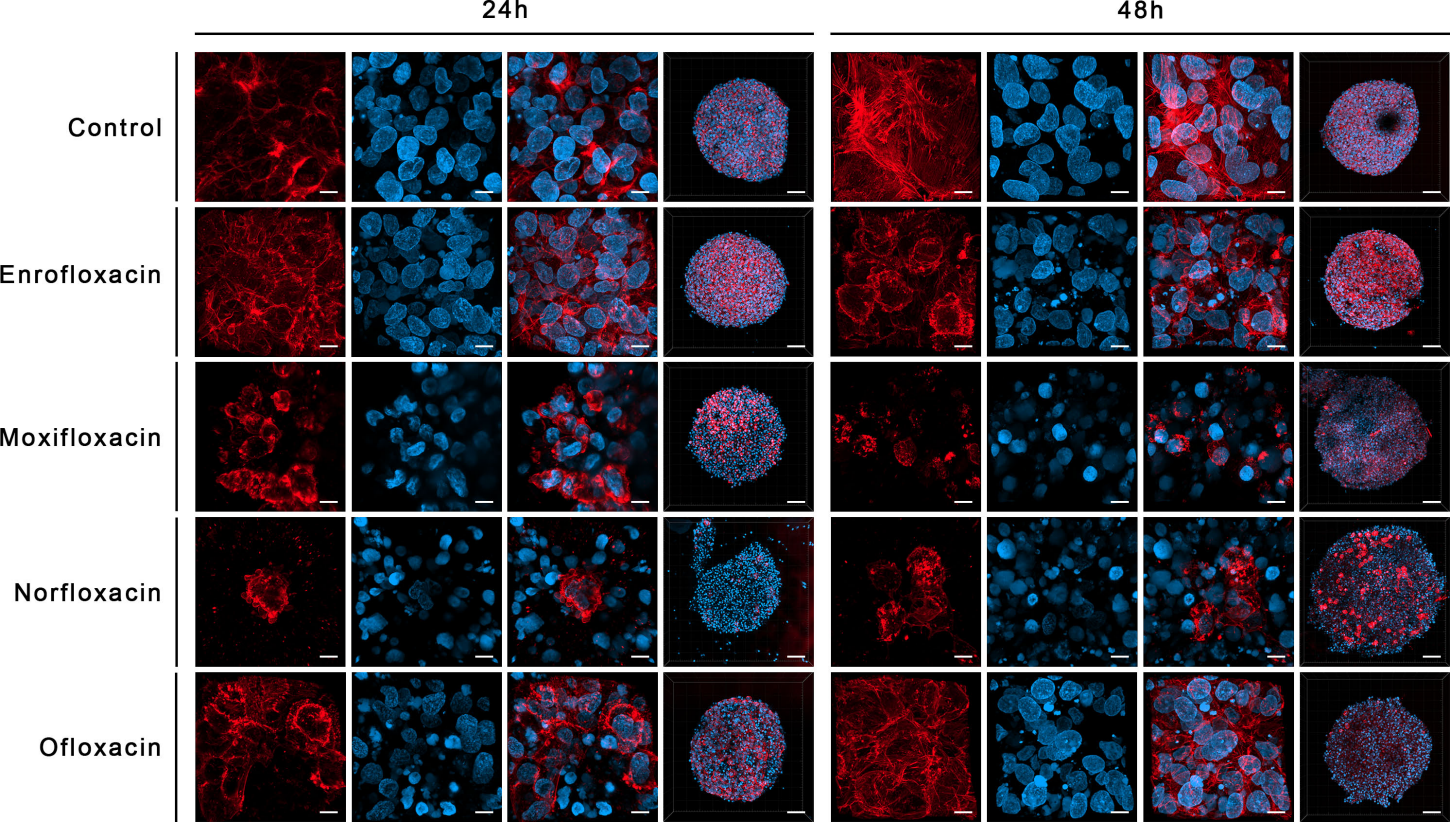
Immunofluorescence staining of cells cytoskeleton in 3D culture in bladder cancer cell line (T24). Results of actin cytoskeleton visualization after exposure cells to LC_90_ concentrations of tested drugs for 24 and 48 hours. Affection of structural integrity, changes in cytoskeletal organization, and nucleus appearance can be observed. Confocal microscope, bar=80 µm and 10 µm for 10x and 63x magnification, respectively.

LC_90_ of all tested compounds affected the structural integrity of the spheroids grown from T24 and SV-HUC1 cell lines. LC_90_ of moxifloxacin had the most severe effect on the cytoskeleton of both cell lines in the 3D spheroid model. Staining revealed a complex degradation of stress fibers at LC_50_ regardless of exposure time. DAPI staining showed a noticeable decrease in nucleus complexity, causing degradation and multiple deformations compared to the control. LC_90_ of enrofloxacin and ofloxacin substantially affected the F-actin cytoskeleton of T24 and SV-HUC-1 spheroids after 24 hours of incubation. Minor differences between these two drugs were visible after 48 hours of incubation, where enrofloxacin had a more significant effect. LC_50_ of enrofloxacin and ofloxacin also caused visible cytoskeleton degradation. This concentration had the most considerable impact on T24 spheroids after 48 hours of incubation. LC_50_ of both drugs affected the nuclei morphology of SV-HUC-1 spheroids strongly after 24 and 48 hours. This effect was also visible for T24 spheroids, but a more decisive response was observed after 48 hours. Norfloxacin caused similar changes in both tested cell lines regardless of exposure. In the case of this drug, we observed more severe nuclei morphology changes than with the other tested drugs. It was evident when imaging SV-HUC-1 cells, where the LC_10_ caused noticeable artifacts of the cell nuclei. On the contrary, norfloxacin did not cause F-actin cytoskeleton changes even at LC_50_ for SV-HUC-1, but we did observe those changes for T24 cell spheroids. The morphological cytoskeleton changes were very pronounced for LC_50_ and LC_90_. Exposure to LC_50_ caused almost no changes in those parameters, whereas LC_90_ caused significant changes (**Figures 9**, **10**; **Supplementary Figures 4**-**7**).

## 4 Discussion

Bladder cancer is one of the most common malignant cancers among men worldwide. Currently, the most common method of treatment for bladder cancer is tumor resection, combined with the prophylactic administration of chemotherapeutics to prevent relapses and metastases. The insufficient effectiveness of these treatment methods and the rising costs of clinical trials have limited the creation of new drugs. With the continuous increase in cancer incidence and treatment costs, the best solution seems to be to repurpose substances already in use today, which is a much cheaper and faster process than *de novo* drug production (2, 6, 9).

Fluoroquinolones are synthetic broad-spectrum antibiotics widely used to treat various infections. Some of these antibiotics show anticancer activity *in vitro* in many cancer cell lines. The anticancer activity of fluoroquinolones is associated with the inhibition of the eukaryotic analogs of DNA gyrase and topoisomerase IIα, however, their effectiveness against eukaryotic topoisomerases is much smaller compared to their prokaryotic counterparts (1000-fold, 24). This indicates that other mechanisms are responsible for fluoroquinolone cytotoxicity in eukaryotic cells, so conducting new experiments with these drugs is important. To our knowledge, this study is the first to compare the effect of 4 different fluoroquinolones on both cancer and normal bladder cells. Additionally, this is the first study analyzing the impact of enrofloxacin, routinely used in veterinary medicine. Such a comparison will allow the determination of the safety and possibility of using these substances in the treatment of bladder cancer (7–9).

Among the fluoroquinolones utilized in this study, only ofloxacin was previously examined against bladder cancer cells. In the first study, the effects of this drug were analyzed on 3 different cell lines (T24, TCCSUP, and J82). The cytotoxic effects of ofloxacin were examined together with the effect of pH on the effectiveness of this antibiotic (6.9–7.2 and around 5.5, which occurs in the urine). The cells were exposed to 0–800 µg/mL concentrations for 24, 48, 72, and 120 hours. It has been shown that acidic pH increases the effectiveness of this drug (proliferation inhibition) from 55.3% to 82.2% for T24, 35.5% to 80.3% for TCCSUP, and 38.4% to 53.6% for J82 in the highest tested doses after 24 hours of incubation. The tested concentrations were the same as in our study. Additionally, the results obtained by Seay et al. are similar to those obtained in our study, as a concentration of 800 µg/mL resulted in a 55.3% inhibition of proliferation, and our calculated LC_50_ was 690 µg/mL (25). A similar study on T24, HTB9, and TCCSUP cell lines also confirmed the effectiveness of ofloxacin against cancer cells. In this study, the drug was incubated with cells for 1, 2, 24, 48, and 96 hours. However, only results after 2 and 96 hours of incubation were presented, so directly comparing our results is difficult. After 96 hours of incubation, concentrations of 150 and 800 µg/mL reduced T24 cell viability by 50% and 90% respectively, indicating that prolonged use of fluoroquinolones can make it possible to obtain effective concentrations in the urine. In this study, tested fluoroquinolones significantly enhanced the cytotoxicity of doxorubicin (26). In another experiment, the effects of ofloxacin on T24 and BOY bladder cancer cell lines were studied. Cells were incubated at 0–500 µg/mL for 24, 48, 72, and 96 hours. Cell growth inhibition was observed depending on fluoroquinolone concentration. No significant changes in the cell cycle were observed at concentrations of 10 and 100 µg/mL after 24-hour incubation. However, a concentration-dependent decrease in telomerase activity was detected (27, 28).

Comparison of the cytotoxic effects of norfloxacin and its gold complex was analyzed against three cancer cell lines A20 (mouse lymphoma), K562 (human myeloma), and B16-F10 (mouse melanoma) and two normal lines L919 (mouse pulmonary fibroblasts) and MCR-5 (human pulmonary fibroblasts). The cells were incubated for 48 hours with concentrations ranging from 0.2–200 µM. The results of this study showed that the norfloxacin-gold (III) complex was more effective (IC_50_ < 65 µM for normal cells and IC_50_ < 55 µM for cancer) than unmodified norfloxacin IC_50_ > 200 µM (~ 63.9 µg/mL for both normal and cancer cell lines). These results are similar to those presented in this study, where the LC_50_ for norfloxacin was 72.4 µg/mL for bladder cancer cells and 129.2 µg/mL for normal urothelial cells (15, 29).

Effect of moxifloxacin was examined against human acute monocytic leukemia (THP-1) and human acute T-cell leukemia (Jurkat) cell lines. Cells were incubated with 5–20 µg/mL of moxifloxacin for 24, 48, and 72 hours. Moxifloxacin at concentrations of 5 and 10 µg/mL did not affect the proliferation of THP-1 and Jurkat cells. Only 20 µg/mL caused a 20 and 24% inhibition of THP-1 and Jurkat cell proliferation after 72 hours of incubation, respectively. It was shown that moxifloxacin in the studied concentrations did not induce apoptosis in THP-1 and Jurkat cells (30). In another study, the effect of moxifloxacin alone and in combination with etoposide on human HT-29 colorectal cancer cells was performed. The experiment showed that cells incubated with moxifloxacin alone did not show proliferation inhibition, and only at concentrations of 20 µg/mL was slight (-11%) inhibition observed. Combining antibiotics with etoposide at 5 µg/mL reduced proliferation by 66%. It was also shown that moxifloxacin significantly increased topoisomerase II inhibition caused by etoposide. Analysis with flow cytometry showed that moxifloxacin did not cause changes in the cell cycle, but the combination with etoposide decreased the number of cells in the G2/M phase and significantly increased the number of cells in the subG1 phase (31). In our study, all tested fluoroquinolones showed an increase in the number of cancer cells in the G2/M phase. In another study, moxifloxacin was added to pancreatic ductal adenocarcinoma cell lines (MIA PaCa-2 and Panc-1). Cells were incubated with the drug at concentrations of 0–400 µg/mL. The study showed dose- and time-dependent inhibition of cell proliferation and cell cycle arrest in the S phase (32). These results indicated that the molecular mechanisms of drug action may differ depending on the cell type and origin.

The effect of enrofloxacin on bladder cancer cells or any other cancer cells has not been studied so far, probably because this fluoroquinolone is commonly used in veterinary medicine to treat urinary and genital infections and has not been used in clinical practice. Enrofloxacin, like other quinolones, has two primary therapeutic targets from the topoisomerase family - DNA gyrase and topoisomerase IV, which are responsible for DNA replication in the bacterial cell (33). Therefore, it can be suspected that, like other fluoroquinolones belonging to this family, it will inhibit the S or G2/M phase of the cell cycle. The results of this study confirmed this hypothesis. Enrofloxacin, in the highest tested concentration (LC_90_), inhibits the cell cycle in phases S (SV-HUC-1) and G2/M (T24) and induces cancer cell death by apoptosis.

Molecular analysis showed that the pro-apoptotic *BAX* gene was overexpressed in non-cancer cell lines treated with moxifloxacin and ofloxacin and in cancer cell lines treated with moxifloxacin. In the case of ofloxacin, increased expression was also observed in T24 cell lines, however, the results were not statistically important. Increased expression of the anti-apoptotic *BCL-2* gene was observed mainly in non-cancer cells. Both genes were overexpressed after treatment with high doses of the tested drugs (LC_90_). Ofloxacin produced the weakest effect on both topoisomerase isoenzymes in the cancer cell lines, while in the case of non-cancer cells, this effect was observed in the *TOP2A* gene of the enrofloxacin group. Results of *CDKN1* expression suggest that in lower concentrations (LC_50_), pro-survival pathways were activated (increased expression), while in higher concentrations (LC_90_), pathways responsible for cell death were initiated (decrease expression or lack of product detection, **Figure 5**). These results are consistent with our previous study (34).

The effect of fluoroquinolones on 3D cultures was, for the first time, evaluated by our group, which analyzed the influence of ciprofloxacin and levofloxacin (34). In this study, similarly, only the highest tested concentrations (calculated LC_90_) were effective against cancer cell lines. It is essential that this effect was practically not visible in non-cancer cell lines (**Figure 6**). Only in the case of moxifloxacin after 24 hours and ofloxacin after 48 hours a decrease in cell viability was observed, which can indicate that *in vivo*, these drugs in high concentration can damage the bladder urothelial layer.

Serum concentrations of fluoroquinolones in human blood are ~100 times lower (3.9 µg/mL for norfloxacin, 5.5 µg/mL for ofloxacin, and 5.0 µg/mL for moxifloxacin) than those that have a significant cytotoxic and pro-apoptotic effect on the T24 cell line. However, the concentration of these antibiotics in target tissues often exceeds the serum concentration. The effectiveness of bladder cancer treatment with fluoroquinolones is highly dependent on the concentration that the drug reaches in the urine because that will be the concentration the cancer cells are in contact with. According to the studies carried out, after a single oral dose of 400 mg of all the fluoroquinolones, norfloxacin reaches the highest concentration in the urine (up to 478 µg/mL) while ofloxacin is next (427 µg/mL), and third is moxifloxacin (127 µg/mL). According to the results obtained in this study, in the case of norfloxacin, only the calculated LC_90_ cannot be achieved in the urine after 24 hours of incubation. In the case of moxifloxacin, LC_50_ values obtained after 48 hours are achievable in the urine, while only LC_10_ was reached after 24 hours of incubation. Ofloxacin has the worst potential, as only LC_10_ values can be achieved in the urine. However, after administration of a four-times higher dose (1600 mg) of norfloxacin, a concentration of 881 µg/mL was recorded in the urine, and in the case of ofloxacin in one examined patient, concentrations reached 1107 µg/mL after a single 400 mg dose. Therefore, it is possible that after using higher fluoroquinolone doses, increased urine concentration can be achieved leading to a more effective inhibition of bladder cancer growth (15–17, 35, 36). Longer incubation with drugs reduces their effective doses, which is why prolonged fluoroquinolone application could result in receiving adequate doses of the drugs in the urine. Because enrofloxacin is a veterinary medicine, its concentrations in urine have not been examined so far.

In urology, the most prescribed fluoroquinolones are ciprofloxacin and levofloxacin (37). Additionally, ciprofloxacin was the most studied fluoroquinolone *in vitro* for its potential anticancer properties (20, 21, 38–40). Between these two drugs, ciprofloxacin has more promising properties due to its higher cytotoxic effects against cancer cell lines and higher concentrations achieved in the urine (1087 µg/mL and 620 µg/mL after administration of 750 mg of ciprofloxacin and levofloxacin, respectively, 36, 41). The disadvantage of ciprofloxacin at high doses is the possibility of crystal nephropathy, for which crystal formation is also visible in *in vitro* cultures (42).

To search for an alternative to ciprofloxacin, we used four different fluoroquinolones in this study, three from the second generation (including one used in veterinary practice) and one from the fourth generation. Besides ciprofloxacin and levofloxacin, ofloxacin, norfloxacin, and moxifloxacin are also often prescribed fluoroquinolones (43, 44). Ofloxacin’s clinical activity is comparable to ciprofloxacin, used mainly for treating respiratory tract, skin, and urinary tract infections. Ofloxacin is used mainly in ear and eye drops as for other indications, more effective and less toxic counterparts replaced it. However, the average adverse effect rate for ofloxacin is 2.4–12.3% which compares favorably to other fluoroquinolones like ciprofloxacin (45). In our study, this fluoroquinolone was the least effective of all four tested drugs on both cell lines, especially after 48 hours of incubation. Although ofloxacin is excreted primarily unchanged in the urine, the achievable concentrations are lower compared to ciprofloxacin and levofloxacin but were comparable to norfloxacin. Nevertheless, only the LC_10_ values calculated in this study for bladder cancer cell lines could be achieved in the urine, limiting the possible use of this drug for bladder cancer treatment. Norfloxacin is a quinoline monocarboxylic acid used mainly for urinary tract infections and prostatitis treatment. This drug is less active and absorbed slightly slower than ciprofloxacin, and is excreted mainly in the urine, increasing its concentration inside the bladder (46). Considering the results obtained in this study and the concentration of this drug achievable in the urine, norfloxacin seems to be the most promising candidate for bladder cancer treatment. Enrofloxacin is similar to the molecular structure of norfloxacin and ciprofloxacin. Additionally, it is metabolized in most species into ciprofloxacin, which can enhance its effectiveness (47). Unfortunately, the pharmacokinetics of this drug in humans has not been evaluated, which does not allow for appropriate conclusions about its suitability in bladder cancer treatment. In the clinicaltrial.gov database, one clinical study (NCT03575312), in which concentration of this fluoroquinolone in the urine and serum was examined in 6 participants after three different routes of administration. However, despite the completion of the study, no results have been published so far. Moxifloxacin has improved activity against gram-positive bacteria and atypical pathogens. It is used mainly for respiratory tract and skin infections. This drug was less effective than norfloxacin and enrofloxacin, however, its cytotoxic effect was stronger than ofloxacin. The main limitation of introducing this drug for bladder cancer treatment is the low concentration that moxifloxacin can achieve in the urine (48).

## 5 Conclusions

In summary, all four fluoroquinolones reduced the viability of the tested cell lines. Comparing direct effectiveness, the most promising properties belong to norfloxacin and enrofloxacin. These two fluoroquinolones exhibit the highest cytotoxic properties against both tested cell lines. In the case of norfloxacin, almost all calculated LC values for the bladder cancer cell line are achievable in the urine. Application of higher concentrations or prolonged use of this drug could enable the achievement of effective concentrations in urine. However, similar to ciprofloxacin, crystal formation in the culture medium can be observed, but in the case of norfloxacin, changes in the kidneys, like acute interstitial nephritis, are very rare (49). Cytotoxic properties of enrofloxacin are promising; additionally, this drug is metabolized to the most effective fluoroquinolone – ciprofloxacin. However, a clinical study analyzing pharmacokinetic properties and percentage of conversion to ciprofloxacin must be performed to determine its potential in cancer treatment. Application of ofloxacin and moxifloxacin are limited by the relatively low concentrations achieved in the urine and the highest cytotoxic effects on non-cancer cells obtained in 3D culture. The advantage of these fluoroquinolones over norfloxacin and enrofloxacin is their lack of crystal generation in cell culture. The proposed use of norfloxacin and enrofloxacin in clinical trials is directly after TURBT. After tumor resection, drugs can be used through intravesical therapy followed by oral administration for several days in conjunction with standard chemotherapy. Such treatment can potentially kill remaining cancer cells, reducing bladder cancer relapses.

## Data availability statement

The original contributions presented in the study are included in the article/**Supplementary Material**. Further inquiries can be directed to the corresponding author.

## Author contributions

Conceptualization: TK. Methodology: TK. Formal analysis: TK. Investigation: TK, KS, ZF, MPa, PD and ŁK. Resources: MPo. and TD. Data curation: TK. Writing – original draft preparation: TK, ZF, DB, MR and KS. Writing – review & editing: MPo. Supervision: MPo and TD. All authors contributed to the article and approved the submitted version.

## Supplementary material

Supplementary Figure 1 Normal and cancer urothelial cell morphology treated with tested fluoroquinolones. T24 and SV-HUC-1 cell lines were incubated with calculated LC values of norfloxacin, enrofloxacin, moxifloxacin, and ofloxacin after 24 and 48 hours. SV-HUC-1 - normal human urothelium; T24 - human bladder cancer; the inverted light microscope, scale bar = 200 µm.

Supplementary Figure 2 Results of real-time cell growth analysis. T24 and SV-HUC-1 cells after 24h and 48h incubation with LC_10_, LC_50_, LC_90_ of norfloxacin, enrofloxacin, moxifloxacin and ofloxacin. Obtained results confirmed the correctness of LC values calculations performed on the basis of MTT assay. SV-HUC-1 - normal human urothelium; T24 - human bladder cancer.

Supplementary Figure 3 Changes in cytoskeleton after fluoroquinolones treatment in 2D culture. Comparisons of T24 and SV-HUC-1 cell morphology in control and LC_10_ concentration. No differences in cell morphology were observed. SV-HUC-1 - normal human urothelium; T24 - human bladder cancer; fluorescence inverted microscope, scale bar = 20 µm.

Supplementary Figure 4 Immunofluorescence staining of non-cancer human urothelium cell line (SV-HUC-1) cytoskeleton in spheroids. Results of actin cytoskeleton visualization after exposure cells to LC_10_, LC_50_, and LC_90_ concentrations of tested drugs for 24 and 48 hours. The affection of structural integrity can be observed. Confocal microscope, bar=80 µm.

Supplementary Figure 5 Immunofluorescence staining of human bladder cancer cell line (T24) cytoskeleton in spheroids. Results of actin cytoskeleton visualization after exposure cells to LC_10_, LC_50_, and LC_90_ concentrations of tested drugs for 24 and 48 hours. Affection of structural integrity can be observed. Confocal microscope, bar=80 µm.

Supplementary Figure 6 Changes in a non-cancer human urothelium cell line (SV-HUC-1) cytoskeleton after fluoroquinolones treatment in 3D culture. Results of actin cytoskeleton visualization after exposure cells to LC_10_, LC_50_, and LC_90_ concentrations of tested drugs for 24 and 48 hours. Changes in cytoskeletal organization and nucleus appearance can be observed. Confocal microscope, bar=10 µm.

Supplementary Figure 7 Changes in human bladder cancer cell line (T24) cytoskeleton after fluoroquinolones treatment in 3D culture. Results of actin cytoskeleton visualization after exposure cells to LC_10_, LC_50_, and LC_90_ concentrations of tested drugs for 24 and 48 hours. Changes in cytoskeletal organization and nucleus appearance can be observed. Confocal microscope, bar=10 µm.
