# Supplementary figures and images for: Effect of four fluoroquinolones on the viability of bladder cancer cells in 2D and 3D cultures
Source: Front Oncol. 2023 Jul 18;13:1222411. doi: 10.3389/fonc.2023.1222411 (PMC10390741; doi:10.3389/fonc.2023.1222411)

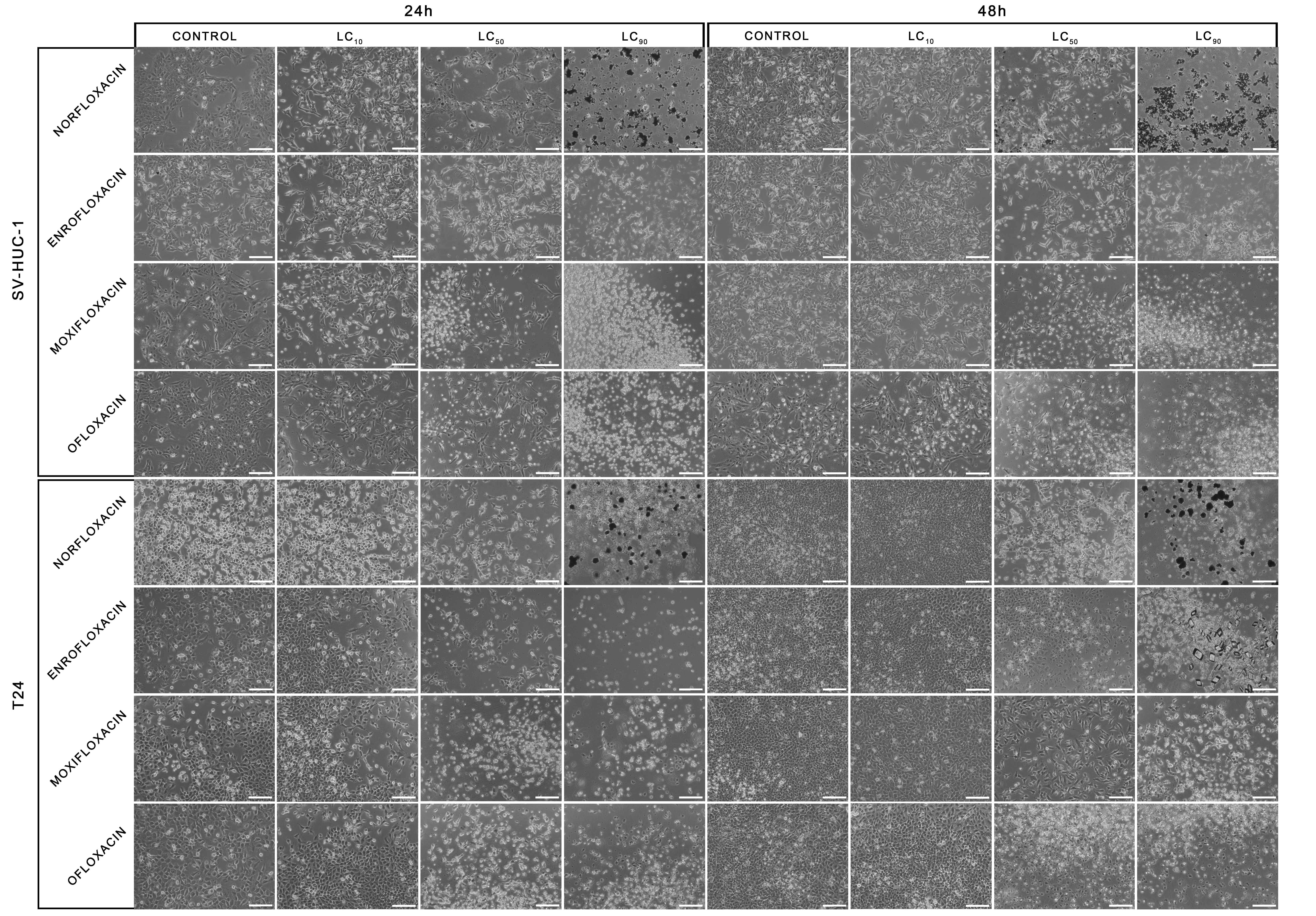

Supplement: Supplementary Figure 1 — Normal and cancer urothelial cell morphology treated with tested fluoroquinolones. T24 and SV-HUC-1 cell lines were incubated with calculated LC values of norfloxacin, enrofloxacin, moxifloxacin, and ofloxacin after 24 and 48 hours. SV-HUC-1 - normal human urothelium; T24 - human bladder cancer; the inverted light microscope, scale bar = 200 µm. [file Image_1.tif]

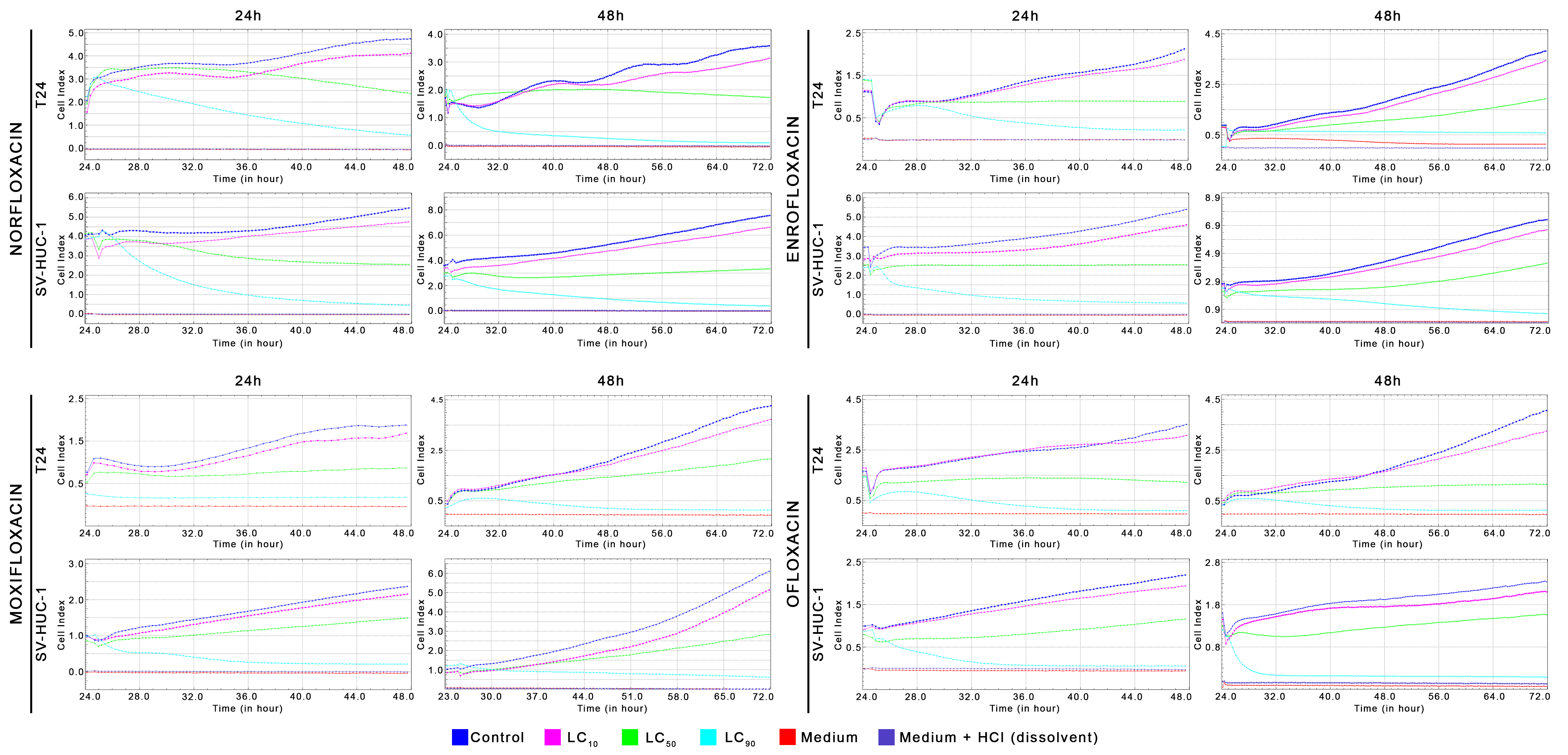

Supplement: Supplementary Figure 2 — Results of real-time cell growth analysis. T24 and SV-HUC-1 cells after 24h and 48h incubation with LC10, LC50, LC90 of norfloxacin, enrofloxacin, moxifloxacin and ofloxacin. Obtained results confirmed the correctness of LC values calculations performed on the basis of MTT assay. SV-HUC-1 - normal human urothelium; T24 - human bladder cancer. [file Image_2.tif]

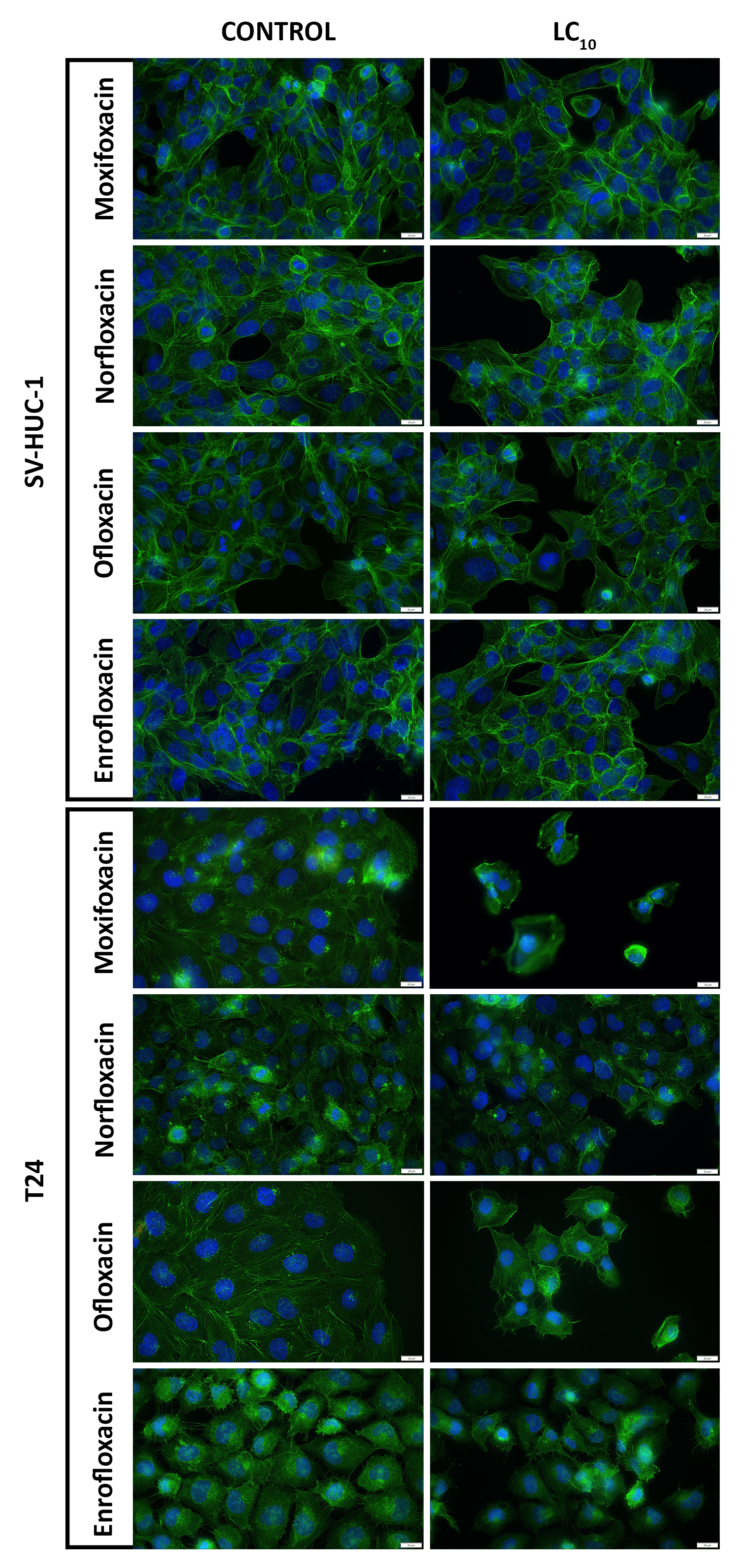

Supplement: Supplementary Figure 3 — Changes in cytoskeleton after fluoroquinolones treatment in 2D culture. Comparisons of T24 and SV-HUC-1 cell morphology in control and LC10 concentration. No differences in cell morphology were observed. SV-HUC-1 - normal human urothelium; T24 - human bladder cancer; fluorescence inverted microscope, scale bar = 20 µm. [file Image_3.tif]

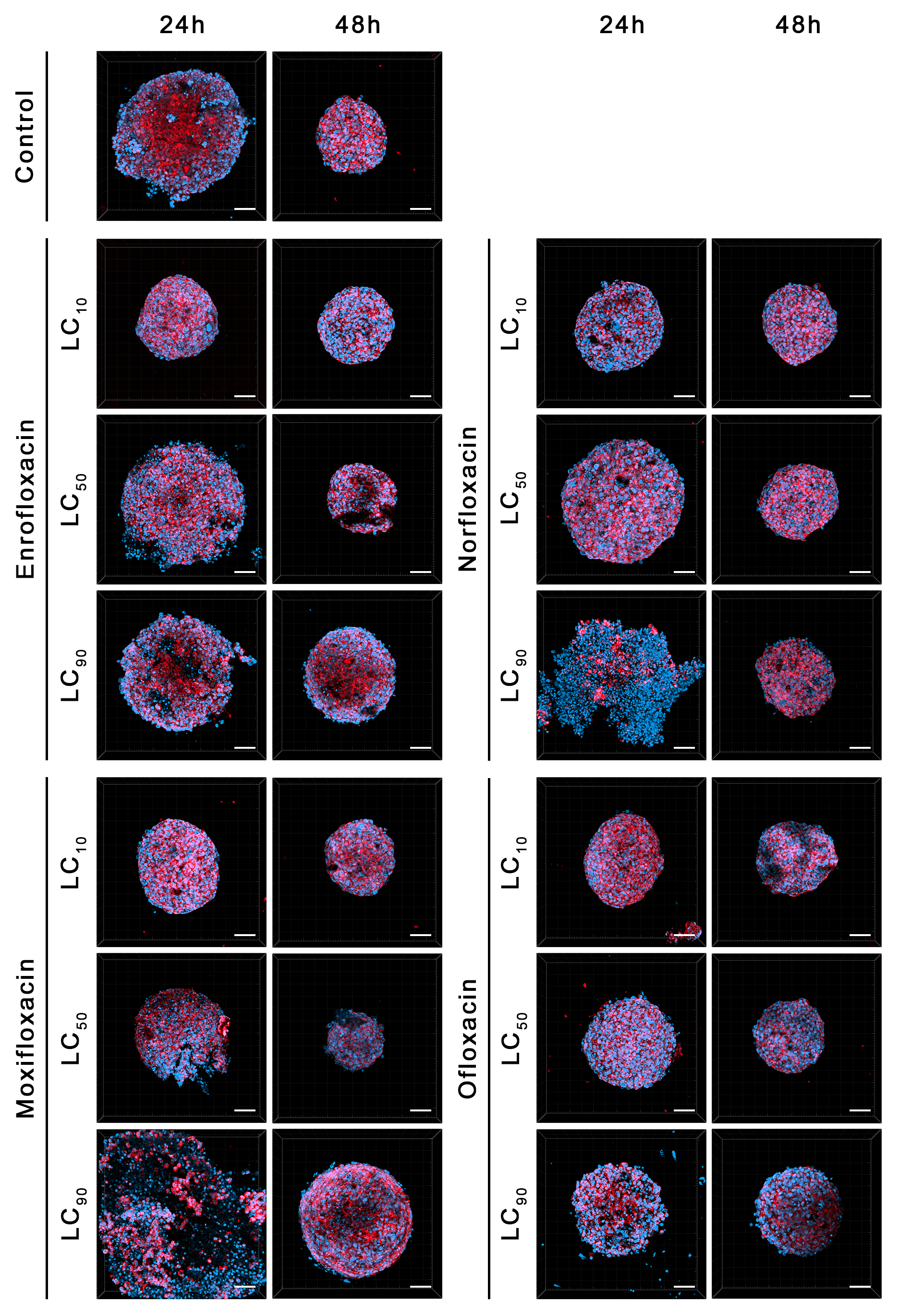

Supplement: Supplementary Figure 4 — Immunofluorescence staining of non-cancer human urothelium cell line (SV-HUC-1) cytoskeleton in spheroids. Results of actin cytoskeleton visualization after exposure cells to LC10, LC50, and LC90 concentrations of tested drugs for 24 and 48 hours. The affection of structural integrity can be observed. Confocal microscope, bar=80 µm. [file Image_4.tif]

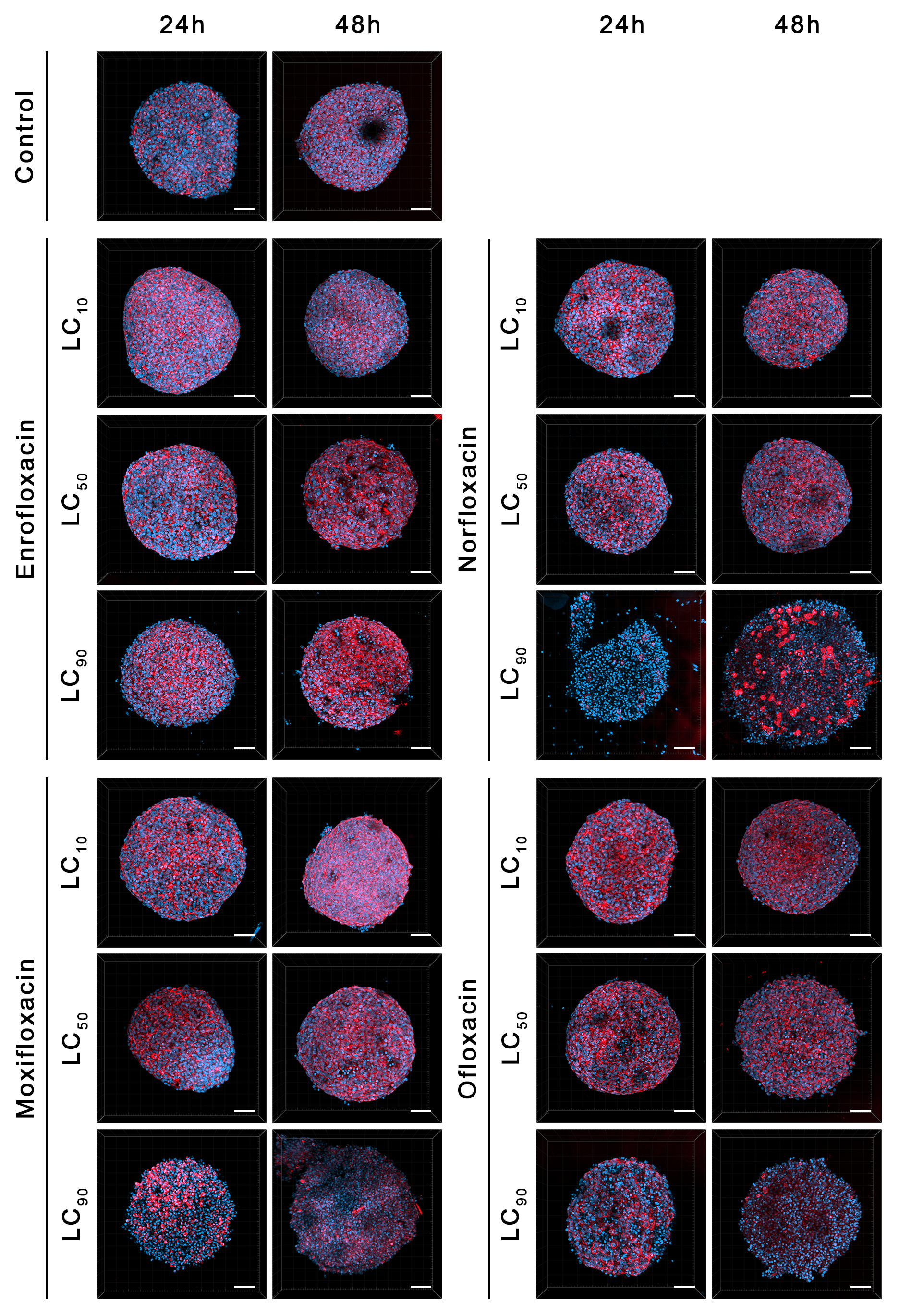

Supplement: Supplementary Figure 5 — Immunofluorescence staining of human bladder cancer cell line (T24) cytoskeleton in spheroids. Results of actin cytoskeleton visualization after exposure cells to LC10, LC50, and LC90 concentrations of tested drugs for 24 and 48 hours. Affection of structural integrity can be observed. Confocal microscope, bar=80 µm. [file Image_5.tif]

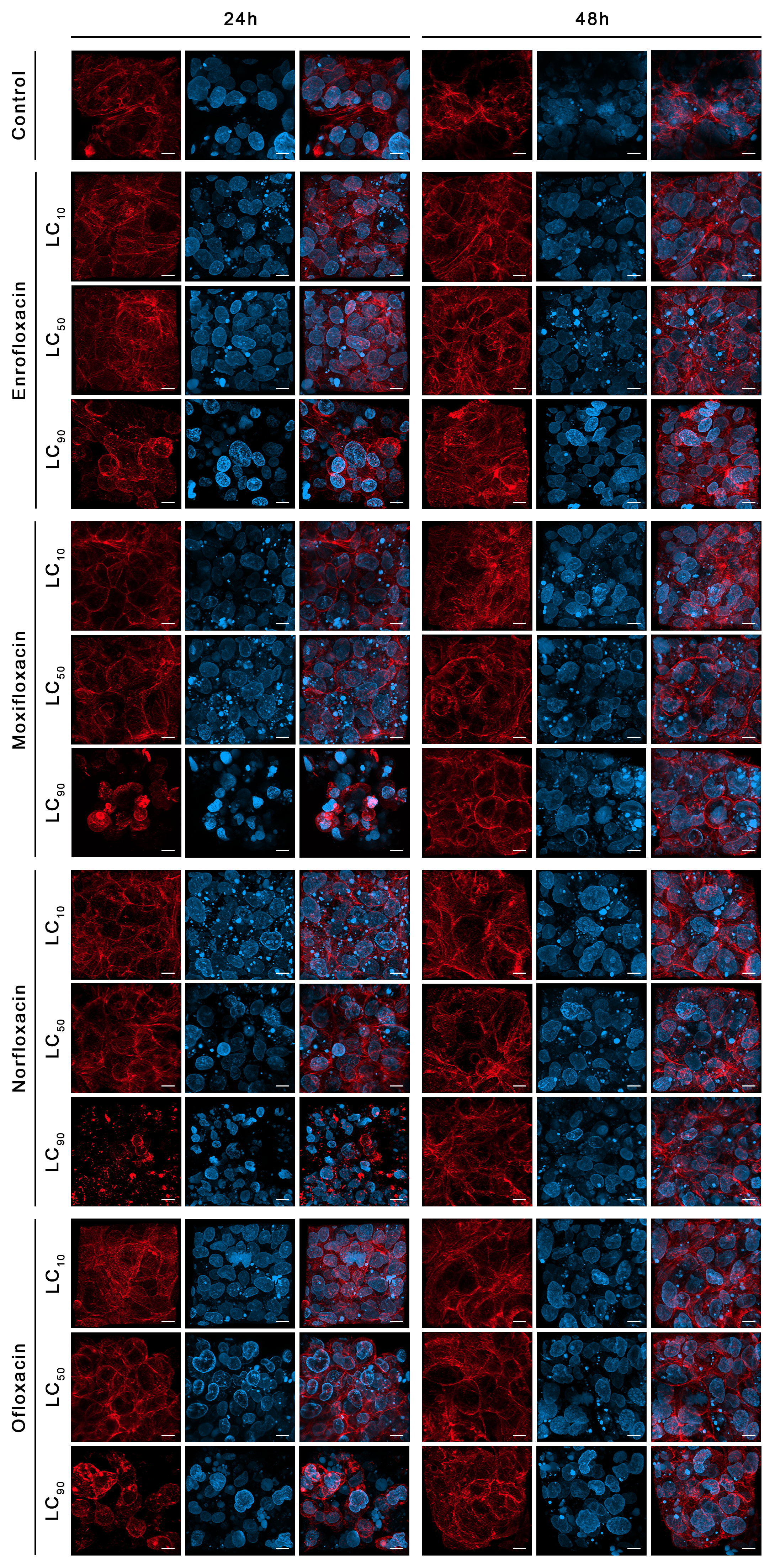

Supplement: Supplementary Figure 6 — Changes in a non-cancer human urothelium cell line (SV-HUC-1) cytoskeleton after fluoroquinolones treatment in 3D culture. Results of actin cytoskeleton visualization after exposure cells to LC10, LC50, and LC90 concentrations of tested drugs for 24 and 48 hours. Changes in cytoskeletal organization and nucleus appearance can be observed. Confocal microscope, bar=10 µm. [file Image_6.tif]

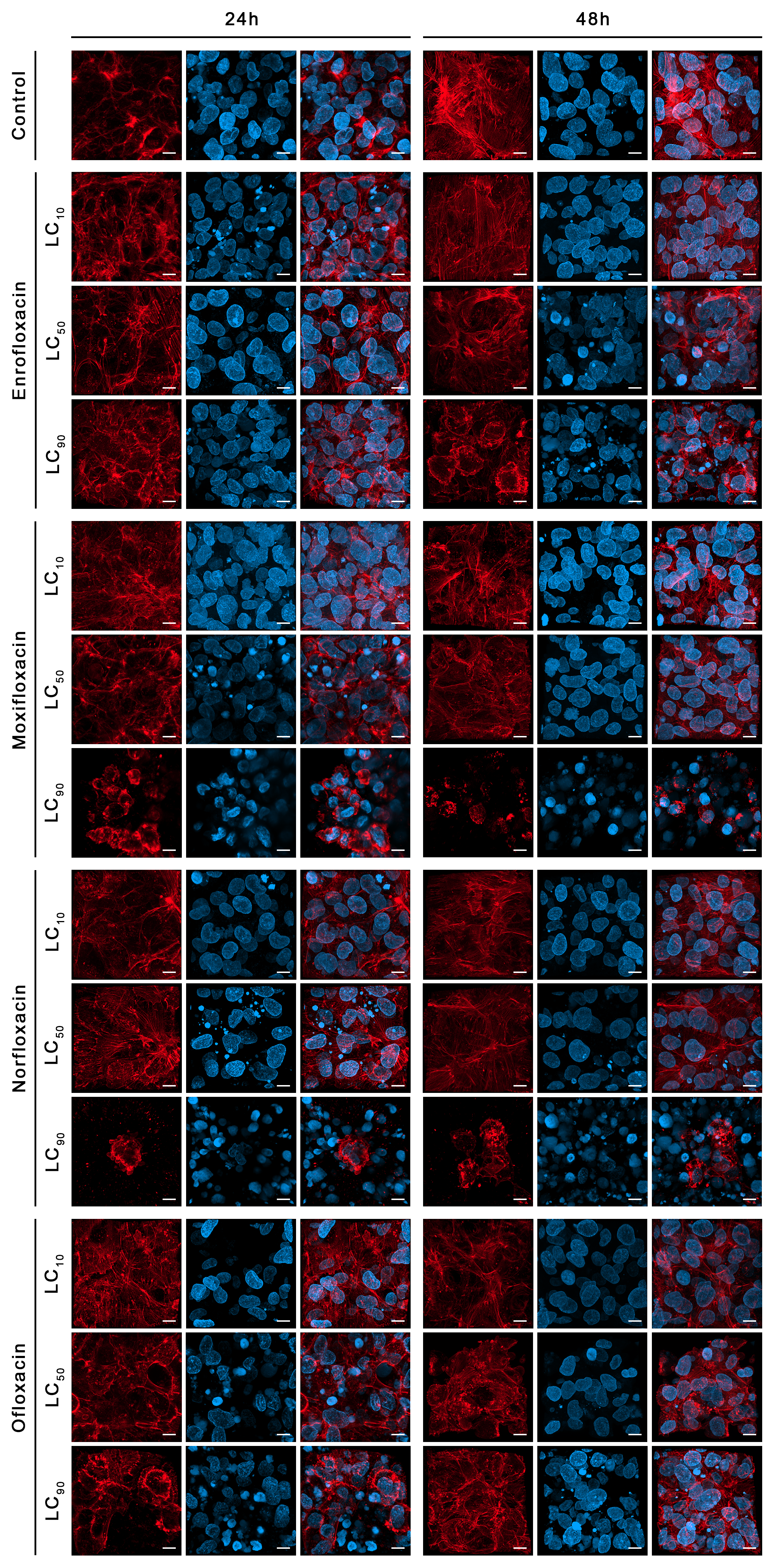

Supplement: Supplementary Figure 7 — Changes in human bladder cancer cell line (T24) cytoskeleton after fluoroquinolones treatment in 3D culture. Results of actin cytoskeleton visualization after exposure cells to LC10, LC50, and LC90 concentrations of tested drugs for 24 and 48 hours. Changes in cytoskeletal organization and nucleus appearance can be observed. Confocal microscope, bar=10 µm. [file Image_7.tif]
