# Supplementary material for: Effect of four fluoroquinolones on the viability of bladder cancer cells in 2D and 3D cultures
Source: Front Oncol. 2023 Jul 18;13:1222411. doi: 10.3389/fonc.2023.1222411 (PMC10390741; doi:10.3389/fonc.2023.1222411)
Supplement: Supplementary file 8 [file Table_1.docx]

**Supp. Tab.1.** Calculated LC values (μg/mL) after incubation of T24 and SV-HUC-1 cell lines with norfloxacin, enrofloxacin, moxifloxacin, and ofloxacin for 24 and 48 hours.

|  |  | **NORFLOXACIN** | | **ENROFLOXACIN** | | **MOXIFLOXACIN** | | **OFLOXACIN** | |
| --- | --- | --- | --- | --- | --- | --- | --- | --- | --- |
|  | **[μg/mL]** | **T24** | **SV-HUC-1** | **T24** | **SV-HUC-1** | **T24** | **SV-HUC-1** | **T24** | **SV-HUC-1** |
| **24h** | **LC_10_** | 21.2 | 26.6 | 22.4 | 18.6 | 20.0 | 33.2 | 105.4 | 41.8 |
|  | **LC_50_** | 146.1 | 208.9 | 179.9 | 185.5 | 430.3 | 301.5 | 693.2 | 430.0 |
|  | **LC_90_** | 1009.2 | 1641.9 | 1444.5 | 1848.0 | 2300.5 | 3042.4 | 2302.6 | 4470.7 |
| **48h** | **LC_10_** | 12.8 | 19.2 | 8.1 | 10.3 | 6.4 | 21.1 | 44.4 | 23.3 |
|  | **LC_50_** | 72.4 | 129.2 | 60.4 | 79.1 | 70.0 | 127.7 | 338.3 | 217.6 |
|  | **LC_90_** | 409.2 | 867.6 | 451.0 | 609.0 | 645.5 | 775.3 | 1143.0 | 1685.9 |
